# Continuous Rating Scale Analytics (CoRSA): A tool for analyzing continuous and discrete data with item response theory

**DOI:** 10.3758/s13428-025-02848-3

**Published:** 2025-11-04

**Authors:** Yeh-Tai Chou, Yao-Ting Sung, Wei-Hung Yang

**Affiliations:** 1 Research Center for Psychological and Educational Testing, National Taiwan Normal University, Taipei, Taiwan; 2 Department of Educational Psychology and Counseling, National Taiwan Normal University, 162, Sec. 1, Ho-Ping E. Road, Taipei, 10644 Taiwan

**Keywords:** Continuous data, Visual analogue scale, Item response model, CoRSA, VAS-RRP

## Abstract

The use of continuous rating scales such as the visual analogue scale (VAS) in research has increased, yet they are less popular than discrete scales like the Likert scale. The non-popularity of continuous scales is primarily due to the lack of validated analytical tools and user-friendly interfaces, which have also jointly resulted in a lack of sufficient theoretical and empirical research supporting confidence in using continuous rating formats. This research aims to address these gaps through four studies. The first study proposed an algorithm and developed the Continuous Rating Scale Analytics (CoRSA) to estimate parameters for the continuous rating scale model (Müller, *Psychometrika*, *52*, 165–181, 1987). The second study evaluated CoRSA’s efficacy in analyzing continuous scores compared to pcIRT (Hohensinn, *Journal of Statistical Software*, *84*, 1–14, 2018) and discrete scores against ConQuest (Adams et al., 2020). Results showed superior parameter recovery with CoRSA for continuous data and comparable outcomes for discrete data. The third study analyzed empirical data from career interest and work value assessments using both VAS and Likert scales with CoRSA, demonstrating good model-data fit and validating CoRSA’s effectiveness in rescaling data to interval measurements. Finally, the fourth study integrated CoRSA into the VAS-RRP 2.0 platform (Sung & Wu, *Behavior Research Methods*, *50*, 1694–1715, 2018) to enhance accessibility and usability, allowing researchers and practitioners unfamiliar with statistical procedures to easily analyze continuous data. These findings confirm CoRSA as a valid tool for analyzing both continuous and discrete data, enhancing the utility of continuous rating formats in diverse research contexts.

**Supplementary Information:**

The online version contains supplementary material available at 10.3758/s13428-025-02848-3.

## Introduction

### Item response theory

#### Continuous response formats

Continuous response formats, in which the respondent’s item responses on a scale are recorded as continuous scores, have received increasing interest in recent years for measuring various psychological (e.g., pain perception) and educational latent traits (e.g., career interest). The most well-known example of continuous response formats is the visual analogue scale (VAS; Hayes & Patterson, 1921), where respondents make a mark along a horizontal or vertical line to express their level of agreement or preference for a latent construct (Couper et al., 2006). The item response is then scored by measuring the distance between the mark and the left endpoint (Reips & Funke, 2008). For example, a respondent may make a mark at the midpoint of the line, and this response might be recorded as 0.50 on a scale ranging from 0 to 1. Continuous response formats can be viewed as an extension of graded response formats, where the number of categories approaches infinity (Mellenbergh, 1994; Müller, 1987; Samejima, 1973). These formats offer more fine-grained measurements (Chimi & Russell, 2009), as well as obtaining test scores with higher variability, enhancing their reliability (Cook et al., 2001; Krieg, 1999) and responsiveness in measuring changes (Pfennings et al., 1995). Recently, the use of continuous response formats has become increasingly prevalent in clinical, educational, and psychological assessments, including domains such as career interest (Sung et al., 2017), depression (May & Pridmore, 2020), mood (Barrows & Thomas, 2018), pain perception (Kersten et al., 2014), personality (Kuhlmann et al., 2017), and quality of life (Feng et al., 2014; Hauser & Walsh, 2008).

#### Item response theory models for continuous responses

Within the item response theory (IRT) framework, several models have been proposed for analyzing continuous responses. These models describe the relationship between the probability density of a specific item response and parameters such as a person's trait level or an item's difficulty. Examples include the continuous response model (CRM; Samejima, 1973), continuous rating scale model (CoRSM; Müller, 1987), linear response model (Mellenbergh, 1994), nonlinear congeneric model (Ferrando, 2001), beta response model (BRM; Noel & Dauvier, 2007), and the zero-and-one inflated models (Molenaar et al., 2022). Molenaar et al. (2022) provided a comprehensive review of these models, with a particular focus on the conditional distributions of responses and the response situations. In the CRM (Samejima, 1973), for instance, it is assumed that an individual's response follows the S_B_ distribution, and individuals can mark any position along a continuous line, except at the endpoints, which is called the open response situation (Samejima, 1973). Consequently, transformations are needed to prevent scores on the scale’s boundaries (Molenaar et al., 2022). A notable feature of the CRM is that the item information is constant across all ability levels (García-Pérez, 2024), implying that the precision of ability estimates remains uniform. The EstCRM R package (Zopluoglu, 2012) is available for conducting parameter estimations using the CRM. In the CoRSM (Müller, 1987), it is assumed that an individual's response follows a truncated normal distribution. Unlike the CRM, the CoRSM allows item information to vary with ability level, enabling differential estimation precision along the ability continuum. In the BRM (Noel & Dauvier, 2007), responses are modeled to follow a beta distribution. BRM focuses on responses in the open interval (0, 1). To compute the log-likelihood effectively, responses at the endpoints of a scale—originally scored as 0 or 1—are typically adjusted to.0001 or.9999, respectively (Noel & Dauvier, 2007). Parameter estimation for the BRM can be performed using either the authors' original software or the sirt R package (Robitzsch, 2024).

Among these three IRT models, the CoRSM offers several distinctive features that make it particularly suitable for applied research. First, the CoRSM permits respondents to mark any point along a continuum, including the endpoints—referred to as the closed response situation (Samejima, 1973). This feature is advantageous because it eliminates the need for data transformations that are required to prevent scores at the scale's boundaries. In contrast, such transformations are required in both the CRM and the BRM when applied to closed response situations, in order to prevent biased parameter estimates (Molenaar et al., 2022). Second, the CoRSM is highly flexible with respect to data types. It extends the rating scale model (RSM; Andrich, 1978), making it capable of accommodating both continuous responses (e.g., markings on a VAS) and discrete responses (e.g., ratings on a Likert-type scale). Müller (1987) demonstrated that the model could analyze four-point discrete responses by approximating the response probability for each category with the corresponding area under a smooth curve (see Fig. 2 in Müller’s original publication). Additionally, as a member of the Rasch model family (Noel & Dauvier, 2007), the CoRSM can transform ordinal item responses into interval-level scores. This transformation is essential for conducting parametric statistical analyses (Embretson, 1996; Harwell & Gatti, 2001; Iramaneerat et al., 2008; see also Wright & Mok, 2004 for an overview of Rasch models). These features are summarized in Table 1, which compares the features of CoRSM with those of the CRM and BRM.

**Table 1:** Comparisons of the features for three continuous IRT models

Model	Response situation	Response distribution	Item information
Continuous response model (CRM; Samejima, 1973)	Open response situation	S_B_ distribution	Remains uniform across different ability levels
Continuous rating scale model (CoRSM; Müller, 1987)	Closed response situation	Truncated normal distribution	Varies with the ability levels
Beta response model (BRM; Noel & Dauvier, 2007)	Open response situation	Beta distribution	Varies with the ability levels

In our view, these features make CoRSM particularly advantageous for researchers and practitioners in applied settings. Accordingly, this study focused on evaluating the performance of the CoRSM—specifically in terms of parameter recovery—across various testing conditions, rather than conducting empirical comparisons with the BRM and CRM. Our goal was to provide practical guidance for researchers who work with both continuous and discrete data formats and who seek to derive interval-level scores suitable for parametric statistical analyses.

#### Barriers to expanding the use of continuous response formats in research and practice

The development of computer-based interfaces has significantly reduced the efforts required for manually processing continuous ratings on paper-based forms—a procedure that is both time-consuming and labor-intensive (Reips & Funke, 2008; Sung & Wu, 2018). Despite this advancement, the adoption of continuous response formats in research and practice still lags far behind that of discrete scales, such as the Likert scale. At least two critical issues need to be addressed to facilitate the broader adoption of continuous response formats.

#### More valid tools for analyzing continuous scores need to be developed for researchers and practitioners

There are abundant on-shelf tools available for conducting IRT analyses, such as ConQuest (Adams et al., 2020), Winsteps (Linacre, 2023), IRTPRO (Cai et al., 2011), and WinBUGS (Spiegelhalter et al., 2003), along with various freely available R packages (Choi & Asilkalkan, 2019). However, it is notable that most of these computer programs are designed for the analysis of discrete data. Only a few, such as WinBUGS, EstCRM (Zopluoglu, 2012), and pcIRT (Hohensinn, 2018), are capable of handling continuous data formats. This scarcity of valid tools for continuous data analysis may hinder the broader adoption of continuous response formats and models. Taking the CoRSM (Müller, 1987) as an example, despite its several distinctive features, the model is not well known by researchers, nor is it commonly used by practitioners. One major factor hampering its adoption is the lack of efficient algorithms and valid analytical tools for parameter estimation. As a member of the Rasch model family, the CoRSM theoretically permits item parameter estimation via the conditional maximum likelihood (CML; Andersen, 1970). However, in practice, this method is hindered by intensive computational demands. Verhelst (2019) discussed these technical challenges in detail, noting that the computational burden of the CML approach renders it largely impractical. Currently, the primary tool available for estimating parameters of the CoRSM is the pcIRT R package (Hohensinn, 2018). This package employs the pairwise conditional likelihood (PAIR; Choppin, 1985; Müller, 1999) method, which has been shown to provide accurate estimates of overall item difficulty in both dichotomous and polytomous Rasch models (Garner & Engelhard, 2002). The robustness of the PAIR approach for CoRSM under diverse testing conditions has not been fully validated. Additionally, pcIRT uses maximum likelihood estimation (MLE) to obtain person parameters. However, because the MLE method yields estimates of negative or positive infinity for examinees who obtain either zero or perfect raw scores, pcIRT does not provide MLE estimates for these two extreme cases.

Another barrier to the broader adoption of the CoRSM is the lack of user-friendly tools accessible to general users, particularly those who require data analysis tools but are not proficient in R programming. Although the pcIRT package implements CoRSM in R, it still requires users to interact with R scripts. For users unfamiliar with the R environment, even basic syntax can present a hurdle to conducting CoRSM analyses. Similarly, while using WinBUGS, another tool that can handle continuous data analyses, users must have knowledge of Bayesian statistical methods and Markov chain Monte Carlo (MCMC) algorithms (Johnson, 2017). These requirements pose a considerable challenge for users without an extensive mathematical background, making the parameter estimation process quite daunting. To enhance the accessibility and utilization of the CoRSM, there is a need to develop more intuitive and user-friendly tools that lower the entry barrier for CoRSM analysis. These tools are specifically designed to support users who either do not use R or lack training in advanced statistical modeling. Simplifying the interface and automating estimation procedures would make analytical tools more accessible to general users in the social sciences and could potentially facilitate the adoption and application of the CoRSM across various fields.

#### More theoretical and empirical research is needed to uncover the psychometric nature of continuous scales

Due to the lack of user-friendly interfaces and easily accessible analytical tools, there is currently limited research investigating the psychometric properties of continuous scales. The first issue of concern revolves around determining the optimal granularity of measurement scales. Researchers are interested in comparing the reliability or measurement errors of fine- and coarse-grained scales, and they hold differing views on the advantages of scales with varying levels of coarseness. For example, some researchers found that coarse-grained measuring scales offer sufficient reliability and that an excessive number of points would create distractions that instead lower the reliability of the test (e.g., Cox, 1980; Tourangeau et al., 2000). However, other researchers hold the view that fine-grained measuring scales provide more options and flexibility, which can subsequently maximize the accuracy of a test (e.g., Cook et al., 2001; Reips & Funke, 2008; Russell & Bobko, 1992). It is noteworthy that, since the continuous scale can be considered as a special case of the discrete scale (Samejima, 1973), comparing the differences between discrete scales with various points as well as continuous scales with infinite points is important to fully answer the above questions. However, instead of collecting truly continuous scores and analyzing the data with appropriate tools for continuous data, all of the research either collected discrete scores or analyzed their data with tools for discrete scores. For instance, Simms et al. (2019) and Flynn et al. (2004) compared the VAS with Likert scales that have response options ranging from 2 to 11 points. In these two studies, the VAS scores were divided into 1000 or seven discrete points. Then the reliability was calculated using the divided scores with a summation function instead of an integration function, which may result in the loss of information provided by continuous response formats and would not accurately reflect the statistical properties of continuous scales.

The second issue of concern is the intervalness of continuous scales. Researchers advocate employing interval scores instead of ordinal scores to avoid the issue of inappropriate use of parametric statistics for ordinal data (Harwell & Gatti, 2001; Iramaneerat et al., 2008; Wright & Mok, 2004) and to improve the responsiveness of change scores (Kersten et al., 2014). Although there is plenty of research and tools addressing the issue of transforming discrete ordinal scores into interval scores (see de Leeuw & Mair, 2007, for the special volume titled ‘Psychometrics in R’), much less research investigates the intervalness of continuous scores. Taking the VAS as an example, some researchers claimed that VAS scores exhibit interval-level measurement properties (Price et al., 1983; Hofmans & Theuns, 2008; Reips & Funke, 2008), while others argue that VAS scores do not behave linearly and lack the property of equal intervals between scores (Kersten et al., 2014). It is interesting to note that none of the research employed methods specifically designed for analyzing continuous scores to substantiate their claims. For instance, Kersten et al. (2014) attempted to compare the intervalness of Likert and VAS scales. In their study, participants marked their pain level on a 10-cm line, which was divided into 50 equal parts. Each mark was scored from 0 to 50. Moreover, the scores were analyzed using a summation function rather than an integration function, which does not correspond with the continuous nature of the scores, potentially compromising the accuracy of reliability calculations. As a member of the family of Rasch models (Müller, 1987; Noel & Dauvier, 2007), the CoRSM can transform ordinal item responses into interval scores. These interval measurements are more suitable for conducting parametric statistical procedures and revealing the intervalness of data derived from continuous scores.

The third issue of interest concerns the relationship between continuous scales and ipsative scores. Researchers have explored the plausibility of integrating ipsative and normative scales using at least two approaches. The first approach is combining the formats of ipsative and normative scales. For example, Chiu and Alliger (1990) proposed the quantitative ranking scale (QRS), which integrates a ranking scale requiring seven items to be ranked and a Likert scale with ten points. Participants can rank the items at any point on the scale, obtaining both ipsative (ranking) and normative (Likert) scores simultaneously. Chiu and Alliger (1990) found that this method performed well in comparison to other scales. Another approach is using statistical methods to transform ipsative scores into normative scores. For example, Brown & Maydeu-Olivares (2011) developed the Thurstonian item response theory (TIRT) model, providing new methods for transforming ipsative ranking data into normative scores. It is notable that when continuous VAS scales are combined with ranking or paired-comparison methods to form the VAS-RRP scales (Sung & Wu, 2018), they become a general case of rating, ranking, and pairwise comparison. This newly proposed technique allows for the simultaneous acquisition of normative scores (rating with continuous scores), ipsative scores (rankings), and partially ipsative/normative scores (rankings with continuous scores). The normative and partially ipsative/normative scores gathered from VAS-RRP scales can be analyzed using continuous IRT models such as the CoRSM mentioned earlier, and the results can be compared with other approaches from Chiu and Alliger (1990) and Brown and Maydeu-Olivares (2011).

The research issues mentioned above represent just a part of the possible issues that need to be addressed by researchers. However, researchers and practitioners need to be empowered by more user-friendly interfaces and valid analytical tools, as well as more research examples, to gain deeper insights and explore the diverse potential usages of continuous scales.

### Purposes of the present article

To address the aforementioned technical and practical issues associated with analyzing continuous rating data within the IRT framework, this research conducted four studies, each with a specific research goal. The first study aims to propose an estimation algorithm for the CoRSM, referred to as Al-CoRSM, utilizing the marginal maximum likelihood (MML; Bock & Aitkin, 1981) and the maximum a posteriori (MAP) methods. In conjunction with this algorithm, we developed a user-friendly analytical tool named Continuous Rating Scale Analytics (CoRSA) to implement Al-CoRSM and support parameter estimation for both researchers and practitioners. The second study aims to verify the capability of CoRSA in analyzing continuous and discrete scores through a series of simulations and to evaluate whether person and item parameters of the CoRSM can be accurately estimated under various measurement conditions. The estimation performances of CoRSA were compared with those obtained using pcIRT (Hohensinn, 2018) and ConQuest (Adams et al., 2020). The third study aims to verify the capability of CoRSA in analyzing two empirical datasets involving continuous and discrete scores and to examine model-data fit for each dataset. This study focuses on demonstrating the applicability of CoRSA in practical applications. The datasets analyzed in this study are available in the Open Science Framework repository at https://osf.io/c3jzy/?view_only=0314d5f0cbef4627812ac0bf5d54d271. The fourth study integrated CoRSA into the VAS-RRP 2.0 platform (Sung & Wu, 2018; http://vasrrp.net/vasrrp2), and focused on introducing its user-friendly interface and functionality. This web-based implementation was developed to serve users who either do not use R or lack training in advanced statistical modeling, enabling them to conduct CoRSM analyses with both continuous and discrete data through an accessible, code-free environment.

## Study 1: Proposing the algorithm for estimating the parameters of the CoRSM

This study proposed an algorithm called Al-CoRSM to estimate the item and person parameters for the CoRSM. Detailed derivations of the MML and the MAP methods as implemented by the Al-CoRSM are then provided, respectively.

### The Continuous Rating Scale Model (CoRSM)

For modeling item responses on a continuous rating scale, Müller (1987) derived a unidimensional IRT model by extending the formulation of the rating scale model (RSM; Andrich, 1978) based on the following assumptions:

1. The rating scale comprises a line of length *L* with midpoint *c*, and
2. The respondent can mark any point along the line in the closed response situation, where the random variable *X* can take any value within the closed interval $x\in \left[c-L/2,c+L/2\right]$.

Let ${x}_{ni}$ refer to the observed response of person *n* responding to item *i* with *n* = 1, 2, …, *N* and *i* = 1, 2, …, *I*. The probability density of ${x}_{ni}$ is given by

1 $$f\left(X=x_{ni}\vert\beta_n,\delta_i,\theta\right)=\frac1{\gamma_1}exp\left[x_{ni}\left(\beta_n-\delta_i\right)+x_{ni}\left(2c-x_{ni}\right)\theta\right]$$

where ${\beta}_{n}$ and ${\delta}_{i}$ denote the locations of person *n* and item *i* on a latent continuum, *θ* is the dispersion parameter, and ${\gamma}_{1}={\int\nolimits_{c-L/2}^{c+L/2}}exp\left[t\left({\beta}_{n}-{\delta}_{i}\right)+t\left(2c-t\right)\theta \right]dt$ is a normalizing factor. When assuming that the person’s rating conforms to a doubly truncated normal distribution, Eq. (1) can be written as

2 $$\left(X=x_{ni}\vert\beta_n,\delta_i,\theta\right)=\frac{exp\left[-\theta\left(x_{ni}-c-\frac{\beta_n-\delta_i}{2\theta}\right)^2\right]}{\int_{c-L/2}^{c+L/2}exp\left[-\theta\left(t-c-\frac{\beta_n-\delta_i}{2\theta}\right)^2\right]dt}$$

The parameter *θ* reflects the shape of the rating distribution (Andrich, 1982). A smaller *θ* indicates that examinees’ item responses are distributed over a wide interval and thus have a larger variance of the item responses, which leads to higher discrimination (Verhelst, 2019). The item characteristic curves of the CoRSM for three *θ* values (e.g., 0.5, 2.0, and 6.0) were presented in Appendix 1. Because the length of the line in the response format can be expressed in arbitrary units and with an arbitrary reference point (Müller, 1987), so that we can assume without loss of generality that c = 0 and $x\in \left[-L/2,L/2\right]$.

### Item parameters

Al-CoRSM used the MML method (Bock & Aitkin, 1981) to estimate the item parameters of the CoRSM. Under the assumption of local independence, the conditional probability density, as a function of the whole set $\Theta$ of ${\beta}_{n}$, ${\delta}_{i}$, and $\theta$ parameters, given the observed response vector ${\mathbf{X}}_{n}$ is

3 $$P\left({\mathbf X}_n\vert\Theta\right)=\prod\limits_{i=1}^If\left(x_{ni}\vert\beta_n,\delta_i,\theta\right)=\prod\limits_{i=1}^I\frac{exp\left[-\theta\left(x_{ni}-\frac{\beta_n-\delta_i}{2\theta}\right)^2\right]}{\int_{-L/2}^{L/2}exp\left[-\theta\left(t-\frac{\beta_n-\delta_i}{2\theta}\right)^2\right]dt}$$

If persons are randomly sampled from a population where the latent trait is distributed according to a density function $g\left(\beta \right)$, the marginal probability density of the ${\mathbf{X}}_{n}$ is

4 $$P\left({\mathbf{X}}_{n}\right)={\int\nolimits}_{-\infty }^{+\infty}\prod\limits_{i=1}^{I}f\left({x}_{ni}|\Theta \right)g\left(\beta \right)d\beta$$

Then the likelihood function is

5 $$L=\prod\limits_{n=1}^{N}P\left({\mathbf{X}}_{n}\right)=\prod\limits_{n=1}^{N}{\int\nolimits}_{-\infty }^{+\infty}\prod\limits_{i=1}^{I}f\left({x}_{ni}|\Theta \right)g\left(\beta \right)d\beta$$

And the log-likelihood function is

6 $$ln\left(L\right)=\sum\limits_{n=1}^{N}ln\left[P\left({\mathbf{X}}_{n}\right)\right]$$

The first partial derivative of Eq. (6) with respect to parameter ${\delta}_{i}$ is

7 $$\frac{\partial ln\left(L\right)}{\partial {\delta }_{i}}=\sum\limits_{n=1}^{N}\frac{1}{P\left({\mathbf{X}}_{n}\right)}\int\nolimits_{-\infty }^{+\infty}\frac{\partial f\left({x}_{ni}\vert\Theta \right)}{\partial {\delta}_{i}}\frac{P\left({\mathbf{X}}_{n}|\Theta \right)}{f\left({x}_{ni}\vert\Theta \right)}g\left(\beta\right)d\beta$$

Equation (7) can be approximated using the Gauss quadrature as follows (see Appendix 2):

8 $$\frac{\partial ln\left(L\right)}{\partial\delta_i}=\sum\limits_{n=1}^N\sum\limits_{q=1}^Q\left(\frac{L_n\left(V_g\right)A\left(V_g\right)}{\sum_{q=1}^{\mathrm Q}L_n\left(V_g\right)A\left(V_g\right)}\frac{\partial f\left(x_{ni}\vert V_g,\delta_i,\theta\right)}{\partial\delta_i}\frac1{f\left(x_{ni}\vert V_g,\delta_i,\theta\right)}\right)$$

An algorithm similar to that described by Roberts et al. (2000) is used to solve these likelihood equations. The values that solve Eqs. (7) and (8) are the MML estimates (${\widehat{\delta }}_{i}$). The Newton–Raphson procedure is used to compute the most likely item parameter estimates for all items. The second partial derivatives needed are as follows (see Appendix 2):

9 $$\frac{{\partial}^{2}ln\left(L\right)}{\partial {\delta}_{i}^{2}}=\sum\limits_{n=1}^{N}\frac{1}{P{\left({\mathbf{X}}_{n}\right)}^{2}}\left[\frac{{\partial}^{2}P\left({\mathbf{X}}_{n}\right)}{\partial {\delta}_{i}^{2}}P\left({\mathbf{X}}_{n}\right)-{\left(\frac{\partial P\left({\mathbf{X}}_{n}\right)}{\partial {\delta}_{i}}\right)}^{2}\right]$$

10 $$\frac{{\partial}^{2}ln\left(L\right)}{\partial {\delta}_{i}\partial {\delta}_{j}}=\sum\limits_{n=1}^{N}\frac{1}{P{\left({\mathbf{X}}_{n}\right)}^{2}}\left(\frac{{\partial}^{2}P\left({\mathbf{X}}_{n}\right)}{\partial {\delta}_{i}\partial {\delta}_{j}}P\left({\mathbf{X}}_{n}\right)-\frac{\partial P\left({\mathbf{X}}_{n}\right)}{\partial {\delta}_{i}}\frac{\partial P\left({\mathbf{X}}_{n}\right)}{\partial {\delta}_{j}}\right)$$

The procedure terminates when the largest change in any ${\widehat{\delta}}_{i}$ from one cycle to the next is less than.001, or until some maximum limit of iterations has been reached (e.g., 25). The initial values of ${\widehat{\delta}}_{i}$ are set to $-\left(\sum\nolimits_{n=1}^{N}{x}_{ni}/N\right)/\!{~}_{{2}\theta}$, which are found to be appropriate to obtain rapid convergence of the estimates of item parameter. Because there is no natural origin in the measurement scale, the mean of all ${\widehat{\delta}}_{i}$ is arbitrarily set to zero.

### Person parameters

Al-CoRSM adopted the MAP method to estimate the person parameters of the CoRSM. The posterior density function of observing a response vector ${\mathbf{X}}_{n}$ is

11 $${f}^{\ast}\left({\beta}_{n}\vert{\mathbf{X}}_{n}\right)=\frac{P\left({\mathbf{X}}_{n}\vert{\beta}_{n}\right)g\left(\beta \right)}{P\left({\mathbf{X}}_{n}\right)}$$

The log posterior function is

12 $$ln{f}^{\ast}\left({\beta}_{n}\vert{\mathbf{X}}_{n}\right)=ln\left[P\left({\mathbf{X}}_{n}\vert{\beta}_{n}\right)\right]+ln\left[g\left(\beta \right)\right]+{\mathrm{constant}}$$

The MAP estimates of person parameters (${\widehat{\beta }}_{n}$) are obtained by setting the first partial derivative of Eq. (12) to zero and solving the equations. The first and second partial derivatives needed for the Newton–Raphson procedure are as follows (see Appendix 2):

13 $$\frac{\partial ln{f}^{\ast}\left({\beta}_{n}\vert{\mathbf{X}}_{n}\right)}{\partial {\beta}_{n}}=\sum\limits_{\mathrm{i}=1}^{\mathrm{I}}\frac{\partial}{\partial {\beta}_{n}}lnf\left({x}_{ni}\vert\Theta \right)-\left(\frac{{\beta}_{n}-\mu}{{\sigma}^{2}}\right)$$

14 $$\frac{{\partial }^{2}ln{f}^{\ast}\left({\beta}_{n}\vert{\mathbf{X}}_{n}\right)}{\partial {\beta}_{n}^{2}}=\sum\limits_{\mathrm{i}=1}^{\mathrm{I}}\frac{{\partial}^{2}}{\partial {\beta}_{n}^{2}}lnf\left({x}_{ni}\vert\Theta \right)-\frac{1}{{\sigma}^{2}}$$

15 $$\frac{{\partial }^{2}ln{f}^{\ast}\left({\beta}_{n}\vert{\mathbf{X}}_{n}\right)}{\partial {\beta}_{n}\partial {\beta}_{m}}=\sum\limits_{\mathrm{i}=1}^{\mathrm{I}}\frac{{\partial}^{2}}{\partial {\beta}_{n}\partial {\beta}_{m}}lnf\left({x}_{ni}\vert\Theta \right)+\frac{\partial}{\partial {\beta}_{m}}\left(-\frac{{\beta}_{n}-\mu}{{\sigma}^{2}}\right)$$

The initial values of ${\widehat{\beta}}_{n}$ were set to $\left({\sum\nolimits}_{i=1}^{I}{x}_{ni}/I\right)/\!{~}_{{2}\theta}$ to obtain rapid convergence.

### Dispersion parameters

Al-CoRSM used the MLE approach to estimate the dispersion parameter. The probability of observing a response matrix $\mathbf{X}$ given ${\boldsymbol{\beta}}$ and ${\boldsymbol{\delta}}$ is equal to

16 $$P\left(\mathbf{X}|\Theta \right)=\prod\limits_{n=1}^{N}\prod\limits_{i=1}^{I}f\left({x}_{ni}|\Theta \right)$$

The log-likelihood function is

17 $$ln\left[P\left(\mathbf{X}|\Theta \right)\right]=\sum\limits_{n=1}^{N}\sum\limits_{i=1}^{I}lnf\left({x}_{ni}|\Theta \right)$$

The MLE estimate of the dispersion parameter ($\widehat{\theta}$) is obtained by using the Newton–Raphson procedure. The MML estimates of item parameters and the MAP estimates of person parameters are used in conjunction with the observed responses to derive $\widehat{\theta }$. The first and second partial derivatives needed are as follows (see Appendix 2):

18 $$\frac{\partial lnP\left(\mathbf{X}|\Theta \right)}{\partial \theta }=\sum\limits_{n=1}^{N}\sum\limits_{i=1}^{I}\frac{\partial}{\partial \theta }lnf\left({x}_{ni}|\Theta \right)=\sum\limits_{n=1}^{N}\sum\limits_{i=1}^{I}\left({A}_{\theta}^{^\prime}-\frac{{\gamma}_{\theta}^{^\prime}}{\gamma}\right)$$

19 $$\frac{{\partial}^{2}lnP\left(\mathbf{X}|\Theta \right)}{\partial {\theta}^{2}}=\sum\limits_{n=1}^{N}\sum\limits_{i=1}^{I}\frac{{\partial }^{2}}{\partial {\theta}^{2}}lnf\left({x}_{ni}|\Theta \right)=\sum\limits_{n=1}^{N}\sum\limits_{i=1}^{I}\left({A}_{\theta}^{^{\prime\prime} }-\frac{{\gamma}_{\theta}^{^{\prime\prime} }\gamma -{{\gamma}^{\prime}}_{\theta }^{2}}{{\gamma}^{2}}\right)$$

## Study 2A: A simulation study with continuous data

The accuracy of parameter estimation is crucial for reliable IRT modeling. Study 2A aimed to evaluate the accuracy of estimation for the CoRSA, which implemented the Al-CoRSM for researchers and practitioners to conduct parameter estimation. The performance of CoRSA was compared to that of pcIRT (Version 0.2.4; Hohensinn, 2019) under different measurement conditions.

### Method

A series of Monte Carlo simulations was conducted to evaluate the accuracy of estimation for person ability, item difficulty, and dispersion parameters under different measurement conditions. The estimated values were compared with the true values of these parameters for simulated continuous data sets. Model parameters were manipulated as described below, using self-written functions and codes in R statistical software (Version 4.1.2) by the authors.

#### Design

Three independent variables were manipulated in this study: sample size (200, 500, and 1000 subjects, indicating small, medium-sized, and large samples, respectively), test length (20, 40, and 60 items, representing short, medium-length, and long tests, respectively), and dispersion parameters (0.5 and 2.0, indicating high and moderate discriminations). We expected that a longer test would improve the person parameter recovery, while a larger sample or a smaller dispersion parameter would improve the item parameter recovery.

Because the pcIRT implicitly rescales the observed data to the unit interval, this leads to the line length changing. Verhelst (2019) demonstrated that rescaling the observed data changes the unit of the latent continuum. An instance of this effect is that halving the observed data will double the values of the person estimates, thereby doubling the mean and standard deviation (SD) of the ability distribution (Verhelst, 2019). Furthermore, since both the MML and MAP methods are usually implemented with the assumption of a normal distribution, it is worthwhile to examine the robustness of these methods when they are applied in the continuous IRT models by comparing the estimation accuracies for normal and nonnormal distributions.

In order to investigate the effect of rescaling the observed variables without modifying the unit of the scale and violating the assumption of a normal ability distribution on parameter recovery, this study took the line length and the form of ability distributions into account jointly in three combinations: (i) the ability distribution being the unit normal *N*(0, 1) and the length of the response scale set as one (denoted as *L* = 1), and thus the lower and upper bounds of the observed variable are defined as 0 and 1, respectively; (ii) the ability distribution being the unit normal and *L* = 5, which means the two bounds are defined as 0 and 5, respectively; and (iii) the ability distribution being nonnormal and *L* = 5. This study focused on examining whether the CoRSA accommodates the unit of the latent scale when rescaling the observed data by comparing the estimation accuracies for normal distributions with different line lengths. There were $3\times 3\times 2\times 3=54$ conditions with 100 replications each.

#### Data generation

To compare the parameter recoveries of the CoRSA and pcIRT, all data sets were simulated using the simCRSM function of the pcIRT (Hohensinn, 2019; retrieved from https://github.com/christinehohensinn/pcIRT/tree/master/R), which generated continuous item responses according to the CoRSM (Müller, 1987). The simCRSM function has five arguments: The first is a vector of item parameters. All generated item difficulties were uniformly distributed within the interval [–2, 2]. The second argument is the value of the dispersion parameter, which was set as 0.5, and 2.0, respectively, for high, and moderate discriminations. The third argument is a vector of person parameters. For the normal distribution, person parameters were randomly sampled from the unit normal distribution *N*(0, 1). For the nonnormal distribution, a polynomial transformation $\beta =a+bx+{cx}^{2}+{dx}^{3}$ was used to transform the person abilities, which leads to the generation of nonnormal ability distributions (Fleishman, 1978). The values of coefficients* a*, *b*, *c*, and *d* were set as – 0.22, 0.78, 0.22, and 0.06, respectively, which resulted in a nonnormal distribution with a mean, SD, kurtosis, and skewness of around 0.04, 1.06, 1.63, and 4.01, respectively. The fourth and fifth arguments of the simCRSM function are the midpoint and length of the response scale, which were set as 0.5 and 1, respectively, for *L* = 1, and as 2.5 and 5 for *L* = 5. The above-described steps were then applied to generate simulated responses for all items answered by all subjects.

All of the simulated data were rescaled to the unit interval and then calibrated using the CoRSA and pcIRT, respectively. The performance in parameter recovery was evaluated by computing the mean absolute deviation (MAD) and root mean square error (RMSE) values for the parameter estimates across 100 replications in each condition. The MAD value was defined as the averaged absolute difference between the estimated and true values of the parameters across all persons or items. The RMSE value was computed by taking the square root of the mean of squared differences between the estimated and true values of the parameter across all persons or items.

Because the unit of the latent trait scale is related to the line length (Müller, 1987), it should be noted that the MAD and RMSE can be compared only when the distributions of true abilities and their corresponding estimated abilities have the same SD. Suppose that simulated continuous item responses were distributed within the interval [0, 5], with the true person abilities following a normal distribution with a mean of zero and a variance of one. Recall that when the item responses are rescaled to the unit interval, which is the implicit procedure implemented by the pcIRT, it can be expected that the estimated person abilities will follow a normal distribution with a mean of zero and a variance of 25. Thus, the estimated person ability estimates need to be divided by 5 to make the SD identical to that for the true person abilities.

### Results

Tables 2, 3, and 4 present the MAD and RMSE values for person ability, item difficulty, and dispersion parameters, respectively, under various testing conditions. A summary of the results is provided below.

**Table 2:** Parameter recovery for person parameters

			Unit normal distribution with line length = 1				Unit normal distribution with line length = 5				Nonnormal distribution with line length = 5			
Independent variables			MAD		RMSE		MAD		RMSE		MAD		RMSE	
Dispersion	Test length	Sample size	CoRSA	pcIRT	CoRSA	pcIRT	CoRSA	pcIRT	CoRSA	pcIRT	CoRSA	pcIRT	CoRSA	pcIRT
0.5	20	200	0.50	0.66	0.63	0.84	0.21	0.63	0.26	0.81	0.21	0.61	0.26	0.79
		500	0.50	0.66	0.63	0.84	0.21	0.66	0.26	0.84	0.22	0.63	0.28	0.87
		1000	0.50	0.66	0.63	0.83	0.21	0.68	0.26	0.87	0.22	0.65	0.29	0.90
	40	200	0.39	0.47	0.49	0.59	0.15	0.67	0.19	0.84	0.15	0.59	0.19	0.76
		500	0.40	0.47	0.50	0.59	0.15	0.71	0.19	0.90	0.15	0.61	0.20	0.84
		1000	0.40	0.47	0.50	0.59	0.15	0.66	0.19	0.83	0.16	0.63	0.21	0.87
	60	200	0.34	0.38	0.42	0.48	0.12	0.69	0.16	0.87	0.12	0.58	0.15	0.75
		500	0.34	0.38	0.43	0.48	0.12	0.67	0.15	0.83	0.13	0.60	0.16	0.83
		1000	0.34	0.38	0.43	0.48	0.12	0.67	0.15	0.84	0.13	0.62	0.17	0.86
2.0	20	200	0.51	0.69	0.64	0.88	0.32	0.72	0.40	0.90	0.32	0.68	0.40	0.87
		500	0.51	0.69	0.65	0.87	0.32	0.74	0.40	0.92	0.32	0.70	0.41	0.93
		1000	0.52	0.69	0.65	0.87	0.32	0.73	0.40	0.91	0.33	0.71	0.42	0.95
	40	200	0.41	0.50	0.51	0.63	0.24	0.73	0.30	0.88	0.24	0.63	0.30	0.80
		500	0.41	0.49	0.52	0.61	0.24	0.68	0.30	0.85	0.24	0.65	0.30	0.88
		1000	0.41	0.48	0.52	0.61	0.24	0.68	0.30	0.86	0.24	0.66	0.30	0.90
	60	200	0.35	0.40	0.44	0.50	0.20	0.65	0.25	0.83	0.20	0.61	0.25	0.78
		500	0.35	0.40	0.44	0.50	0.20	0.68	0.25	0.85	0.20	0.63	0.25	0.85
		1000	0.35	0.40	0.44	0.50	0.20	0.68	0.25	0.85	0.20	0.65	0.25	0.88

**Table 3:** Parameter recovery for item parameters

			Unit normal distribution with line length = 1				Unit normal distribution with line length = 5				Nonnormal distribution with line length = 5			
Independent variables			MAD		RMSE		MAD		RMSE		MAD		RMSE	
Dispersion	Test length	Sample size	CoRSA	pcIRT	CoRSA	pcIRT	CoRSA	pcIRT	CoRSA	pcIRT	CoRSA	pcIRT	CoRSA	pcIRT
0.5	20	200	0.21	0.21	0.26	0.26	0.07	0.85	0.09	0.99	0.07	0.86	0.09	1.00
		500	0.13	0.13	0.16	0.17	0.05	0.85	0.06	0.98	0.05	0.85	0.06	0.98
		1000	0.09	0.10	0.12	0.12	0.04	0.84	0.05	0.98	0.04	0.85	0.05	0.98
	40	200	0.21	0.21	0.26	0.27	0.07	0.84	0.09	0.97	0.07	0.84	0.09	0.97
		500	0.13	0.13	0.17	0.17	0.04	0.83	0.06	0.96	0.04	0.83	0.06	0.96
		1000	0.09	0.09	0.12	0.12	0.03	0.82	0.04	0.95	0.04	0.82	0.04	0.95
	60	200	0.21	0.21	0.26	0.27	0.07	0.83	0.09	0.97	0.07	0.83	0.09	0.97
		500	0.13	0.14	0.17	0.17	0.04	0.82	0.06	0.95	0.04	0.82	0.05	0.95
		1000	0.09	0.10	0.12	0.12	0.03	0.82	0.04	0.95	0.03	0.82	0.04	0.94
2.0	20	200	0.21	0.22	0.27	0.28	0.11	0.86	0.14	1.00	0.11	0.86	0.14	1.00
		500	0.14	0.14	0.17	0.18	0.08	0.85	0.10	0.98	0.08	0.85	0.10	0.98
		1000	0.10	0.10	0.12	0.13	0.06	0.84	0.08	0.97	0.06	0.85	0.08	0.98
	40	200	0.22	0.22	0.27	0.27	0.11	0.84	0.14	0.98	0.12	0.84	0.14	0.98
		500	0.14	0.14	0.17	0.17	0.07	0.83	0.09	0.96	0.07	0.83	0.09	0.96
		1000	0.10	0.10	0.12	0.12	0.05	0.82	0.07	0.95	0.05	0.83	0.07	0.95
	60	200	0.22	0.22	0.27	0.28	0.11	0.83	0.14	0.97	0.11	0.83	0.14	0.97
		500	0.14	0.14	0.17	0.18	0.07	0.82	0.09	0.95	0.07	0.82	0.09	0.95
		1000	0.10	0.10	0.12	0.12	0.05	0.82	0.06	0.95	0.05	0.82	0.07	0.95

**Table 4:** Parameter recovery for dispersion parameters

			Unit normal distribution with line length = 1				Unit normal distribution with line length = 5				Nonnormal distribution with line length = 5			
Independent variables			MAD		RMSE		MAD		RMSE		MAD		RMSE	
Dispersion	Test length	Sample size	CoRSA	pcIRT	CoRSA	pcIRT	CoRSA	pcIRT	CoRSA	pcIRT	CoRSA	pcIRT	CoRSA	pcIRT
0.5	20	200	0.18	0.18	0.23	0.23	0.02	0.49	0.02	0.49	0.01	0.49	0.02	0.49
		500	0.17	0.10	0.20	0.13	0.02	0.48	0.02	0.48	0.02	0.48	0.02	0.48
		1000	0.17	0.09	0.20	0.11	0.02	0.48	0.02	0.48	0.02	0.48	0.02	0.48
	40	200	0.12	0.14	0.15	0.17	0.01	0.49	0.01	0.49	0.01	0.49	0.01	0.49
		500	0.12	0.08	0.15	0.10	0.01	0.49	0.01	0.49	0.01	0.48	0.01	0.48
		1000	0.11	0.06	0.13	0.08	0.01	0.48	0.01	0.48	0.01	0.48	0.01	0.48
	60	200	0.12	0.11	0.14	0.14	0.01	0.49	0.01	0.49	0.01	0.49	0.01	0.49
		500	0.09	0.07	0.11	0.09	0.01	0.48	0.01	0.48	0.01	0.48	0.01	0.48
		1000	0.08	0.05	0.09	0.06	0.01	0.48	0.01	0.48	0.01	0.48	0.01	0.48
2.0	20	200	0.21	0.18	0.25	0.23	0.06	1.95	0.07	1.95	0.05	1.95	0.06	1.95
		500	0.18	0.13	0.22	0.15	0.07	1.93	0.07	1.93	0.07	1.93	0.08	1.93
		1000	0.19	0.08	0.21	0.10	0.07	1.92	0.07	1.92	0.08	1.93	0.09	1.93
	40	200	0.15	0.15	0.17	0.18	0.03	1.95	0.04	1.95	0.03	1.95	0.04	1.95
		500	0.12	0.07	0.15	0.09	0.04	1.93	0.04	1.93	0.04	1.93	0.04	1.93
		1000	0.12	0.06	0.14	0.08	0.04	1.93	0.04	1.93	0.05	1.93	0.05	1.93
	60	200	0.11	0.12	0.14	0.15	0.02	1.95	0.03	1.95	0.02	1.95	0.03	1.95
		500	0.10	0.07	0.12	0.08	0.03	1.93	0.03	1.93	0.03	1.93	0.03	1.93
		1000	0.09	0.05	0.11	0.06	0.03	1.93	0.03	1.93	0.03	1.93	0.03	1.93

#### Effect of sample size

For item parameters, the MAD and RMSE values obtained from CoRSA decreased as the sample size increased, while holding the dispersion parameter constant. This indicates that CoRSA achieved better estimation precision with larger samples. In contrast, the MAD and RMSE values from pcIRT remained relatively unchanged across different sample sizes when line length = 5, suggesting limited sensitivity to sample size in item parameter estimation. Moreover, the effect of sample size on the estimation accuracy of ability and dispersion parameters was negligible for both CoRSA and pcIRT.

#### Effect of test length

For ability and dispersion parameters, the MAD and RMSE values from CoRSA decreased as test length increased when the dispersion parameter was held constant. In contrast, the pcIRT was less sensitive to changes in test length when line length = 5, with minimal or no change in MAD and RMSE values for ability and dispersion parameters. The effect of test length on item difficulty parameter estimation was negligible for both the CoRSA and pcIRT.

#### Effect of dispersion parameter

For the ability, item, and dispersion parameters, the MAD and RMSE values from CoRSA increased as the true dispersion parameter increased. In contrast, the pcIRT was less sensitive to changes in dispersion, with noticeable increases in MAD and RMSE values only for higher dispersion in the estimation of dispersion parameters when line length = 5. For ability and item parameters, the changes were minimal.

#### Effect of data transformation (L = 1 vs. L = 5)

The value of *L* had opposite effects on CoRSA and pcIRT. For all three types of parameters, CoRSA showed lower MAD and RMSE values when *L* = 5 compared to *L* = 1. This was because item responses were concentrated within a narrow interval at *L* = 1 (as shown in subplot C of Fig. 9 in Appendix 1), resulting in lower discrimination and reduced estimation precision. In contrast, pcIRT yielded lower MAD and RMSE values when *L* = 1 compared to *L* = 5, which was particularly evident for item and dispersion parameters. This was primarily due to the pcIRT routinely rescaling the input data to the unit interval without modifying the unit of the trait scale, leading to discrepancies between the estimated and true parameters when *L* = 5.

#### Effect of ability distribution (normal vs. nonnormal)

For all three types of parameters, the estimation accuracy of CoRSA and pcIRT remained robust when the ability distribution deviated from normality. For *L* = 5, the MAD and RMSE values for ability, item difficulty, and dispersion parameters under a nonnormal distribution were comparable to those under a normal distribution. However, pcIRT consistently exhibited higher MAD and RMSE values than CoRSA across all parameter types, regardless of distributional form.

In general, CoRSA provided higher estimation accuracy than pcIRT for all three types of parameters, particularly under conditions where no transformation was applied (e.g., *L* = 5). The accuracy advantage of CoRSA was most pronounced in scenarios with longer tests and larger samples. Conversely, when transformation was applied (e.g., *L* = 1), pcIRT delivered comparable performance to CoRSA for item parameters and slightly outperformed CoRSA in estimating dispersion parameters.

For researchers and practitioners who seek better model–data fit rather than interval-level parameter estimates, a slight extension of Müller’s model proposed by Verhelst (2019) can be used to analyze data. Verhelst’s model incorporates both item difficulty and item-level dispersion for each item. To address this need, we derived the estimation algorithm for Verhelst’s model and conducted simulations under the same testing conditions identical to Study 2A, with one exception: the dispersion parameters for each item were randomly sampled from the interval [0.3, 2.0], thereby mimicking items with moderate and high discrimination. The MAD and RMSE values under each condition are presented in Appendix 3. The patterns of parameter recovery were similar to those reported in Tables 2, 3, and 4. Overall, the algorithm for Verhelst’s model demonstrated satisfactory accuracy in estimating person abilities, item difficulties, and item-level dispersions. However, performance declined under the conditions where the ability distribution was normal and the line length = 1.

#### Estimation efficiency of the CoRSA and pcIRT

Table 5 presents the computation time (in seconds) required to complete the parameter estimation procedures for both CoRSA and pcIRT. For example, under the condition with a dispersion of 0.5, a test length of 60, and a sample size of 200, CoRSA completed the estimation in 12.4 s, whereas pcIRT required 74.8 s, which was approximately six times longer. This pattern was consistent across most conditions, except for two cases involving a dispersion value of 2.0, a test length of 20, and a sample size of 1000. Overall, these results indicate that CoRSA is more computationally efficient than pcIRT for parameter estimation. All analyses were conducted on a desktop computer with an Intel Core i5-14500 processor (2.60 GHz), 32 GB of RAM, running Windows 10.

**Table 5:** Estimation efficiency of the CoRSA and pcIRT

Independent variables			Unit normal distribution with line length = 1		Unit normal distribution with line length = 5		Non-normal distribution with line length = 5	
Dispersion	Test length	Sample size	CoRSA	pcIRT	CoRSA	pcIRT	CoRSA	pcIRT
0.5	20	200	3.1	9.0	5.5	12.9	4.7	12.9
		500	5.5	16.7	12.5	23.9	13.0	24.4
		1000	11.4	23.4	25.3	33.4	25.4	34.1
	40	200	6.7	34.5	12.1	51.1	9.8	50.8
		500	13.3	63.6	24.5	93.6	24.9	94.3
		1000	23.1	86.7	45.2	128.3	49.6	129.5
	60	200	12.4	74.8	19.7	114.8	20.6	113.8
		500	23.7	136.7	39.3	209.6	39.3	209.9
		1000	40.8	187.1	79.4	284.9	86.5	285.6
2.0	20	200	4.3	9.0	9.2	15.2	20.7	34.0
		500	8.4	16.6	18.9	22.7	25.0	30.0
		1000	15.1	23.3	35.1	27.9	37.3	31.9
	40	200	9.8	35.1	20.8	61.0	21.1	63.2
		500	18.6	64.3	41.2	90.2	43.3	97.7
		1000	33.7	88.0	76.6	108.8	81.4	116.8
	60	200	17.7	78.5	37.9	137.5	38.5	145.5
		500	34.0	143.3	73.8	202.6	79.4	216.0
		1000	60.6	194.7	137.9	242.4	354.3	554.2

## Study 2B: A simulation study with discrete data

Since continuous item responses can be regarded as a limiting case of discrete item responses in which the number of response categories becomes infinitely large (Mellenbergh, 1994; Samejima, 1973), the present study postulated that the CoRSA can also be used to perform parameter estimations for discrete data when the distances between two adjacent step difficulty parameters are equal (this is the case 3 of the rating scale model, Andrich, 1978). This study aimed to recover the parameters using the CoRSA by comparing the estimated values with the generated true values of the ability, overall difficulty, and step difficulty parameters for simulated discrete data sets. Parameter recovery in ConQuest (Adams et al., 2020) was used for comparison purposes as appropriate.

### Method

Monte Carlo simulations were used to evaluate parameter recovery under different measurement conditions. To simulate 100 data sets under each condition, discrete item responses were generated according to the RSM (Andrich, 1978). The probability function of the discrete response is given by

20 $${P}_{nik}=\frac{exp\left[\sum\limits_{j=0}^{k}\left[{\beta}_{n}-\left({\delta}_{i}+{\tau}_{j}\right)\right]\right]}{\sum\limits_{k=0}^{m}exp\left[\sum\limits_{j=0}^{k}\left[{\beta}_{n}-\left({\delta }_{i}+{\tau}_{j}\right)\right]\right]},{\mathrm{where}}\sum\limits_{j=0}^{0}\left[{\beta}_{n}-\left({\delta}_{i}+{\tau}_{j}\right)\right]\equiv 0$$

where *P*_*nik*_ is the response probability of scoring *k* on item *i* for subject *n*, *β*_*n*_ is the location of the *n*th person on the continuum, *δ*_*i*_ is the overall difficulty of the *i*th item on the continuum, *τ*_*j*_ is the step difficulty of the *j*th threshold on the continuum, and* m* is the number of response categories in each item. Model parameters were manipulated as described below, using self-written functions and codes in R statistics software (Version 4.1.2).

#### Design

The sample size was fixed at 500 subjects to ensure a sufficient number of subjects for parameter estimation. The number of response categories was set at three levels (five, seven, and nine, which represented three types of commonly used items in discrete rating scales). The test length was set at four levels (5, 10, 20, and 30, which were considered very short, short, medium, and long tests, respectively). Two analytical tools (CoRSA and ConQuest) were used to calibrate the simulated data. In summary, this simulation experiment had 24 (3 × 4 × 2) unique conditions, and 100 replications were carried out under each condition. We expected that the parameter recoveries of the CoRSA would be accurate and similar to those obtained using ConQuest.

#### Data generation

The data generation process in each condition consisted of the following steps: First, ability parameters were randomly generated from the unit normal distribution. Second, overall difficulty parameters for all items were set to range uniformly from – 2.0 to 2.0. Third, the step parameters were set to range from – 2.3 to 2.5. The step parameters were set to the four values of – 2.3, – 0.9, 0.7, and 2.5 when there were five response categories, to – 2.3, – 1.3, – 0.5, 0.2, 1.4, and 2.5 when there were seven response categories, and to – 2.3, – 1.6, – 0.8, – 0.3, 0.1, 0.9, 1.5, and 2.5 when there were nine response categories. Fourth, these generated parameters (abilities, overall difficulties, and step difficulties) were used to compute the corresponding category and cumulative probabilities using the RSM (Andrich, 1978), which is demonstrated in Eq. (20). Fifth, the cumulative probability values were compared with a random number sampled from the uniform distribution (0, 1). The simulated discrete item response was defined as the category with the highest score at which the random number was less than or equal to the corresponding cumulative probability. The above-mentioned steps were performed to generate simulation data for the responses by all subjects on all the items.

Each simulated data set was calibrated using the CoRSA and ConQuest. To evaluate the parameter recovery, the MAD and RMSE values of all estimates for the ability, overall difficulty, and step difficulty parameters were computed, respectively, and were averaged across replications within each condition. This study represented the comparison of the means of the MAD and RMSE values for person, overall difficulty, and step difficulty parameters between conditions.

### Results

Table 6 presents the MAD and RMSE values for person ability, overall item difficulty, and step difficulty parameters under various testing conditions.

**Table 6:** Parameter recovery of the CoRSA and ConQuest

Response category	Test length	Analytical tool	Person parameter		Overall difficulty parameter		Step difficulty parameter	
			MAD	RMSE	MAD	RMSE	MAD	RMSE
5	5	CoRSA	0.470	0.563	0.207	0.214	0.171	0.176
		ConQuest	0.476	0.574	0.201	0.212	0.109	0.127
	10	CoRSA	0.371	0.448	0.202	0.209	0.127	0.130
		ConQuest	0.382	0.461	0.201	0.210	0.099	0.110
	20	CoRSA	0.300	0.363	0.200	0.209	0.101	0.104
		ConQuest	0.312	0.375	0.200	0.209	0.085	0.093
	30	CoRSA	0.276	0.331	0.200	0.209	0.100	0.102
		ConQuest	0.287	0.343	0.200	0.209	0.077	0.083
7	5	CoRSA	0.399	0.481	0.203	0.208	0.202	0.182
		ConQuest	0.408	0.495	0.201	0.210	0.202	0.202
	10	CoRSA	0.318	0.384	0.200	0.206	0.141	0.145
		ConQuest	0.329	0.395	0.201	0.208	0.111	0.125
	20	CoRSA	0.273	0.324	0.200	0.205	0.132	0.135
		ConQuest	0.282	0.333	0.200	0.207	0.116	0.125
	30	CoRSA	0.253	0.298	0.200	0.205	0.108	0.111
		ConQuest	0.266	0.310	0.200	0.206	0.075	0.083
9	5	CoRSA	0.333	0.405	0.201	0.206	0.147	0.151
		ConQuest	0.344	0.416	0.203	0.212	0.122	0.145
	10	CoRSA	0.263	0.319	0.200	0.205	0.143	0.146
		ConQuest	0.275	0.330	0.202	0.208	0.125	0.140
	20	CoRSA	0.220	0.264	0.200	0.204	0.121	0.124
		ConQuest	0.233	0.276	0.200	0.205	0.076	0.087
	30	CoRSA	0.200	0.237	0.200	0.204	0.121	0.123
		ConQuest	0.216	0.254	0.200	0.205	0.037	0.045

#### Effect of response categories

For ability parameters, the MAD and RMSE values obtained from CoRSA decreased as the number of response categories increased when the test length was held constant. This indicates that CoRSA achieved better estimation precision for person ability with more response categories. In contrast, the MAD and RMSE values for the step difficulty parameters slightly increased with more response categories. For example, with a test length of 20, the RMSE increased from 0.104 with five response categories to 0.135 with seven. This suggested that CoRSA provided slightly lower estimation precision for step parameters when tests involved a greater number of response categories. The effect of response category on overall difficulty parameter estimation was negligible, as the MAD and RMSE values remained relatively stable across conditions. ConQuest demonstrated similar patterns to CoRSA across all testing conditions.

#### Effect of test length

For both ability and step difficulty parameters, the MAD and RMSE values obtained from CoRSA decreased as the test length increased when the number of response categories was held constant. This indicates that CoRSA achieved better estimation precision with longer tests. In contrast, the test length had minimal impact on the estimation accuracy of the overall difficulty parameter. Again, ConQuest exhibited similar trends to those observed for CoRSA.

Overall, when CoRSA was used to analyze discrete data with five, seven, and nine response categories, the accuracy of parameter estimation was highly comparable to that achieved by ConQuest. Both tools demonstrated appropriate parameter recovery and were able to accurately estimate ability, overall difficulty, and step difficulty parameters.

#### Summary of Study 2 A and 2B

The simulation studies conducted in Study 2A and 2B provide robust evidence that CoRSA demonstrates effective parameter recovery across various testing conditions. It is observed that increasing the sample size leads to reduced estimation errors for item difficulties, while longer tests decrease estimation errors for person abilities. It is also observed that increasing dispersion parameters diminishes the accuracy of estimations for both person abilities and item difficulties. Additionally, the simulations also highlighted challenges with the pcIRT (Hohensinn, 2018), particularly its lower estimation accuracy when rescaling the observed variables without modifying the unit of the latent scale (e.g., *L* = 5). This limitation hinders the practical applicability of pcIRT. In contrast to pcIRT, CoRSA provided satisfactory parameter estimates even under conditions such as narrow response intervals (e.g., *L* = 1), showcasing its robustness. Finally, the results suggest that CoRSA performs comparably to ConQuest (Adams et al., 2020) when applied to discrete data, indicating its versatility and effectiveness across different types of data.

## Study 3A: Applying the CoRSA to empirical continuous data from the career interest assessment

Studies 3 A and 3B were designed to employ the CoRSA to transform both ordinal continuous and discrete data into interval scores. The Rasch model and its extensions, such as the RSM (Andrich, 1978), are widely recognized for their ability to convert ordinal discrete data into interval scores when data adequately fit the model (Bond & Fox, 2015). As a member of the Rasch family models (Noel & Dauvier, 2007), the CoRSM is similarly expected to be capable of transforming not only ordinal continuous data but also ordinal discrete data into interval scores when data fit this model. Specifically, Study 3A utilized the CoRSA to estimate both person and item parameters, and to evaluate the fit between the CoRSM and the empirical continuous data from the career interest assessment using item fit statistics (Wright & Masters, 1982). When all items demonstrate acceptable fit statistics, it robustly indicates that the data fit the model well. Consequently, the resulting estimates of person abilities and item difficulties are regarded as being on an interval measurement scale (Bond & Fox, 2015; Smith, 2000; Wright & Mok, 2004). These interval-level measures fulfill the requirements of conducting parametric statistical analyses (Harwell & Gatti, 2001; Iramaneerat et al., 2008; Wright & Mok, 2004). It is inappropriate to directly compare the model-data fit of the CoRSA and pcIRT (Hohensinn, 2018) using the empirical dataset, as the true values of the person, item, and dispersion parameters are unknown. Therefore, pcIRT was not utilized in the analysis of the empirical data.

### Method

#### Participants

For this study, the sample comprised 800 ninth graders from junior-high-schools in Taiwan, with a mean age of 13.71 years. Among them were 257 males (32.13%) and 543 females (67.88%). These students took the career interest assessment (see the next section) during career guidance classes, and the results would be used as one of the references for students’ career decisions when considering further streaming to vocational high schools.

#### Assessment tools

**Fig. 1:**
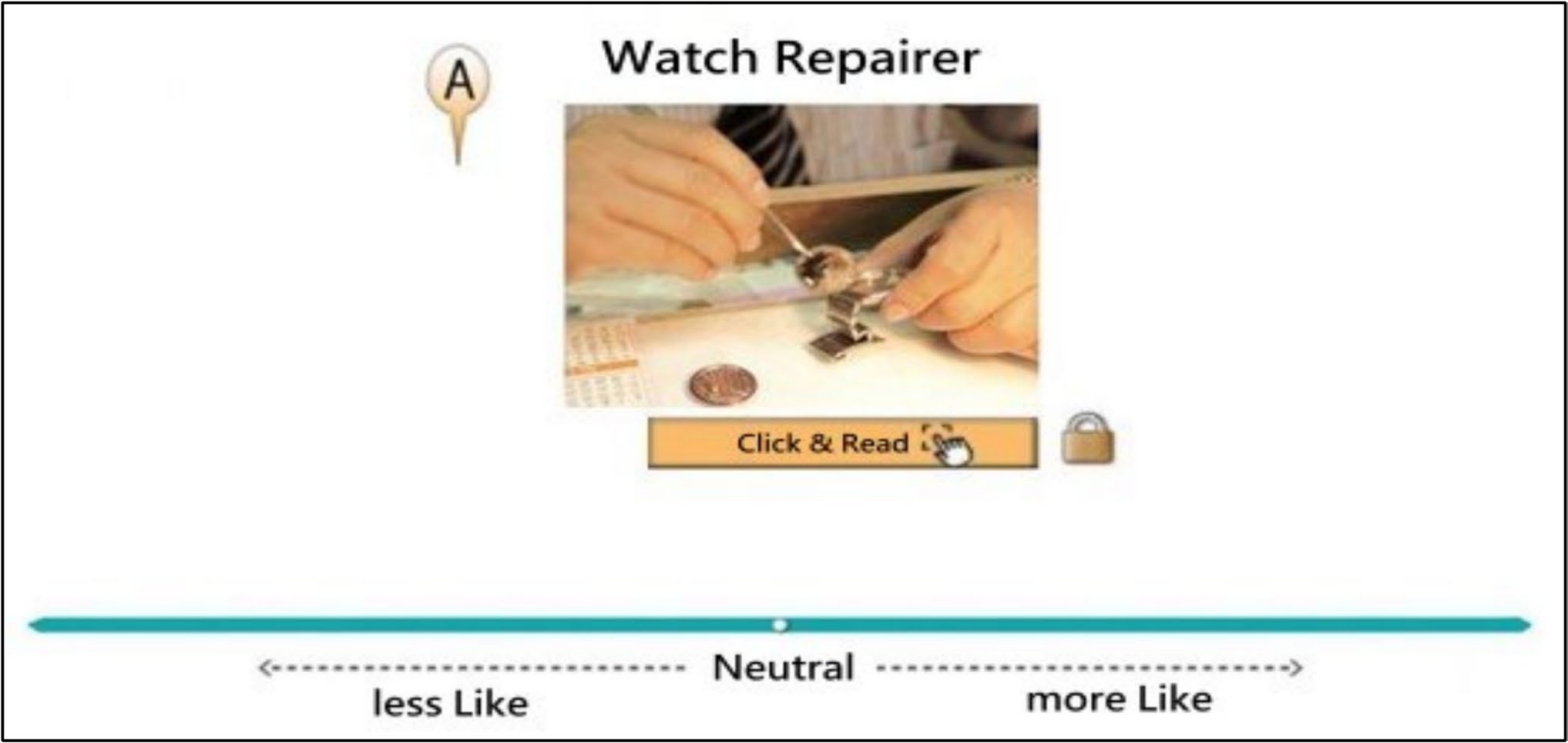
A Screenshot of the Situation-Based Career Interest Assessment (SCIA) item. *Note.* Icon A represents the single description related with the Realistic type of vocational interest

The Situation-Based Career Interest Assessment (SCIA) (Sung et al., 2016, 2017), which is a VAS-format scale, was used to collect participants’ continuous item responses. The SCIA is a computerized test of vocational interest that is designed to help students in grades 7 through 12 with their career choices and is based on the vocational interest theory of Holland (1997), in which vocational interests are categorized into six types: Realistic (R), Investigative (I), Artistic (A), Social (S), Enterprise (E), and Conventional (C). The SCIA included a total of 138 items, with 23 items in each of the six dimensions. Each VAS item comprised an 800-pixel continuous line along with an item describing a job title, a curriculum course, or an activity related to one of the six types of vocational interest (as shown in Fig. 1). Considering that high school students might not be familiar with all of the vocations presented, a picture and description were provided under the heading of each vocation. Students could also click on the icon next to each vocation to learn more about it. As each item appeared on the screen, the student dragged and dropped each vocational description onto the line continuum. Placing a vocational item further to the right of the line indicated that the student had a stronger preference for it. The student could place the item at any point along the line. The ratio of the distance (calculated in pixels) of the item from the left end of the line determined the student’s score for that vocation.

#### Procedure

The test data were collected in April 2022. The purpose of the SCIA and the method of endorsing the item were explained to the participating students before the scale was administered to them. The administration of the SCIA lasted for around 20–25 min.

#### Analysis

To verify the model-data fit of each SCIA dimension, we used the CoRSA to analyze each dimension of the data individually. The raw continuous data were transformed into the unit interval. The outfit mean square error (MNSQ; Wright & Masters, 1982) values for each item in each dimension were calculated as follows:

21 $${\mathrm{MNSQ}}_{i}=\sum\limits_{n=1}^{N}{\left(\frac{{x}_{ni}-{p}_{ni}}{\sqrt{{V}_{ni}}}\right)}^{2}\bigg{/}{N=\sum\limits_{n=1}^{N}{z}_{ni}^{2}}\bigg{/}N$$

where *x*_*ni*_, *p*_*ni*_, *V*_*ni*_, and *z*_*ni*_ are the observed score, expected score, variance of the observed scores, and standardized residual on item *i* for subject *n*, respectively, and *N* is the sample size. The term *V* reflects the spread of the rating distribution. A larger value of *V* indicates that examinees’ item responses are distributed over a wide interval; conversely, a smaller value indicates a more concentrated response pattern. Wright and Linacre (1994) recommended that acceptable MNSQ values fall between 0.6 and 1.4. However, as noted in the same article, Linacre emphasized that low MNSQ values (i.e., overfit) are generally less problematic than high values (i.e., underfit). He advised that when analyzing data from an existing test, items with very low MNSQ values should not be removed, as they can still provide useful information. Therefore, in line with Linacre’s guidance, we retained items with MNSQ values below 0.6 and excluded only those exceeding 1.4. The data were considered to fit the CoRSM adequately if the outfit MNSQ values of all retained items were at or below 1.4 (Bond & Fox, 2015).

### Results

There were 6, 2, 3, 0, 4, and 2 items excluded from the R, I, A, S, E, and C dimensions of the SCIA, respectively. To verify the stability of the model-data fit, we split the full sample into two random subsamples and refitted the model separately to each subsample. The procedures were repeated ten times, and we recorded the number of times each item was classified as fitting (e.g., MNSQ < = 1.4) or misfitting (e.g., MNSQ > 1.4). In the first subsample, for example, within the R dimension, six items (item 3, 7, 12, 19, 22, and 23) were identified as fitting in fewer than seven out of the ten replications. This result indicated that these items consistently misfit the model. Similarly, 2, 3, 0, 4, and 2 items were consistently identified as misfitting in the I, A, S, E, and C dimensions, respectively. Notably, the misfitting items in the first subsample matched those identified in the full sample. The second subsample produced comparable results, again identifying 6, 2, 3, 0, 4, and 2 misfitting items across the R, I, A, S, E, and C dimensions, respectively.

**Fig. 2:**
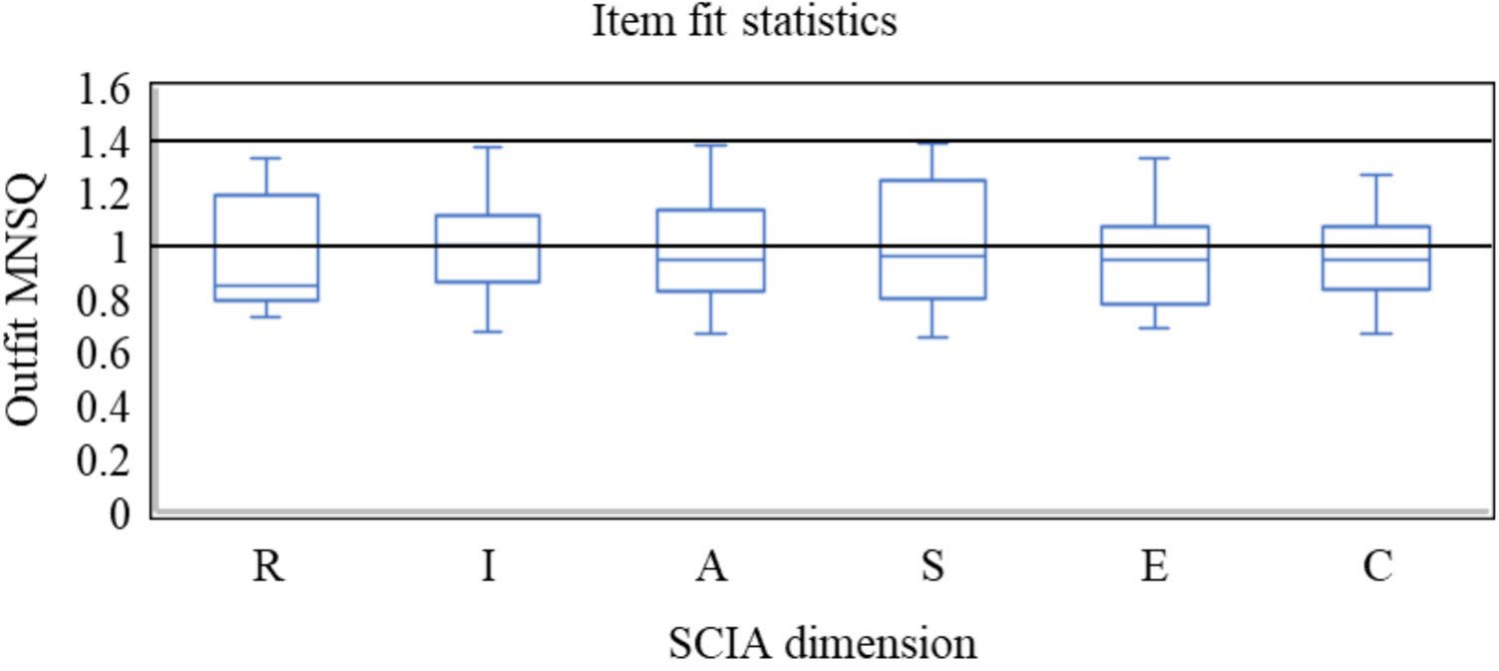
Outfit MNSQ values for remaining items in each SCIA dimension. *Note*. Each box plot shows the median, first and third quartiles, and range. R = Realistic; I = Investigative; A = Artistic; S = Social; E = Enterprise; and C = Conventional

Figure 2 shows the distribution of MNSQ values for items retained in each dimension, where the MNSQ values ranged from 0.66 to 1.39. In addition, the mean MNSQ value ranged between 0.96 and 1.00 for each dimension. These results suggest that the subjects’ responses to the retained items fit the CoRSM well. The dispersion parameter for each dimension was 0.42, 0.41, 0.35, 0.37, 0.53, and 0.58, respectively. Estimates of item difficulty parameters of the remaining items are reported in Table 7. The values of item difficulties across all dimensions ranged from – 0.56 to 0.54. The standard errors of item difficulties ranged between 0.19 and 0.24.

**Table 7:** Results from a CoRSM analysis of the Situation-Based Career Interest Assessment (SCIA)

Item No.	Item difficulty parameter estimate					
	Realistic	Investigative	Artistic	Social	Enterprise	Conventional
1	0.23	– 0.11	– 0.27	0.10	– 0.19	0.21
2	0.15	0.08	-	0.38	0.48	0.54
3	-	– 0.05	– 0.07	0.01	– 0.37	– 0.01
4	0.03	– 0.12	– 0.11	0.08	– 0.26	0.13
5	– 0.18	0.02	0.10	– 0.10	– 0.06	– 0.09
6	– 0.56	0.02	0.23	– 0.20	0.06	0.25
7	-	0.36	-	0.13	– 0.23	0.20
8	0.33	-	0.10	– 0.12	0.03	0.15
9	– 0.12	0.49	– 0.08	– 0.41	-	0.29
10	0.03	– 0.01	– 0.25	– 0.16	– 0.07	– 0.09
11	– 0.34	– 0.15	0.13	0.22	0.26	– 0.05
12	-	0.31	– 0.03	– 0.04	– 0.08	-
13	– 0.08	– 0.01	– 0.32	0.21	0.04	– 0.07
14	0.19	– 0.11	– 0.05	0.20	0.14	– 0.18
15	– 0.03	– 0.01	0.04	0.03	-	– 0.40
16	– 0.03	– 0.11	– 0.23	0.07	– 0.37	– 0.34
17	– 0.17	– 0.14	0.46	0.12	0.09	0.14
18	0.30	0.05	0.27	–0.28	-	– 0.09
19	-	– 0.03	-	– 0.10	0.15	0.01
20	0.24	– 0.24	0.00	0.14	0.04	–0.34
21	0.01	-	– 0.15	– 0.38	0.45	– 0.08
22	-	– 0.25	– 0.01	0.16	– 0.12	– 0.18
23	-	0.01	0.24	– 0.06	-	-
*M*	0.00	0.00	0.00	0.00	0.00	0.00
*SD*	0.23	0.19	0.20	0.20	0.24	0.23

*Note*. “-” indicates that the item was removed and thus the estimate was not available

In summary, the results of the analyses demonstrated that after excluding the poorly fitting items, the remaining items fit the CoRSM well. Based on the well model-data fit, the CoRSA was capable of estimating the interest measures in each dimension for each subject. These estimates conform to the requirements of the interval-level measurement scale for applying parametric statistics (Bond & Fox, 2015; Harwell & Gatti, 2001; Iramaneerat et al., 2008; Wright & Mok, 2004), thereby facilitating more precise and reliable measurement of career interest.

## Study 3B: Applying the CoRSA to empirical discrete data of work value assessment

Study 3B aimed to employ the CoRSA to transform ordinal discrete data into interval scores. Specifically, this study utilized the CoRSA to estimate both person and item parameters, and to evaluate the fit between the CoRSM and the empirical discrete data from the work value assessment using item fit statistics (Wright & Masters, 1982). When all items fit the model well, the resulting estimates of person abilities and item difficulties are on an interval measurement scale (Bond & Fox, 2015; Smith, 2000; Wright & Mok, 2004).

### Methods

#### Participants

For this study, the sample comprised 716 senior-high-school students in Taiwan, with a mean age of 16.92 years. Among them were 354 males (49.44%) and 362 females (50.56%). The results would be used as one of the references for students’ career decisions of entering departments of colleges.

#### Assessment tool

**Fig. 3:**
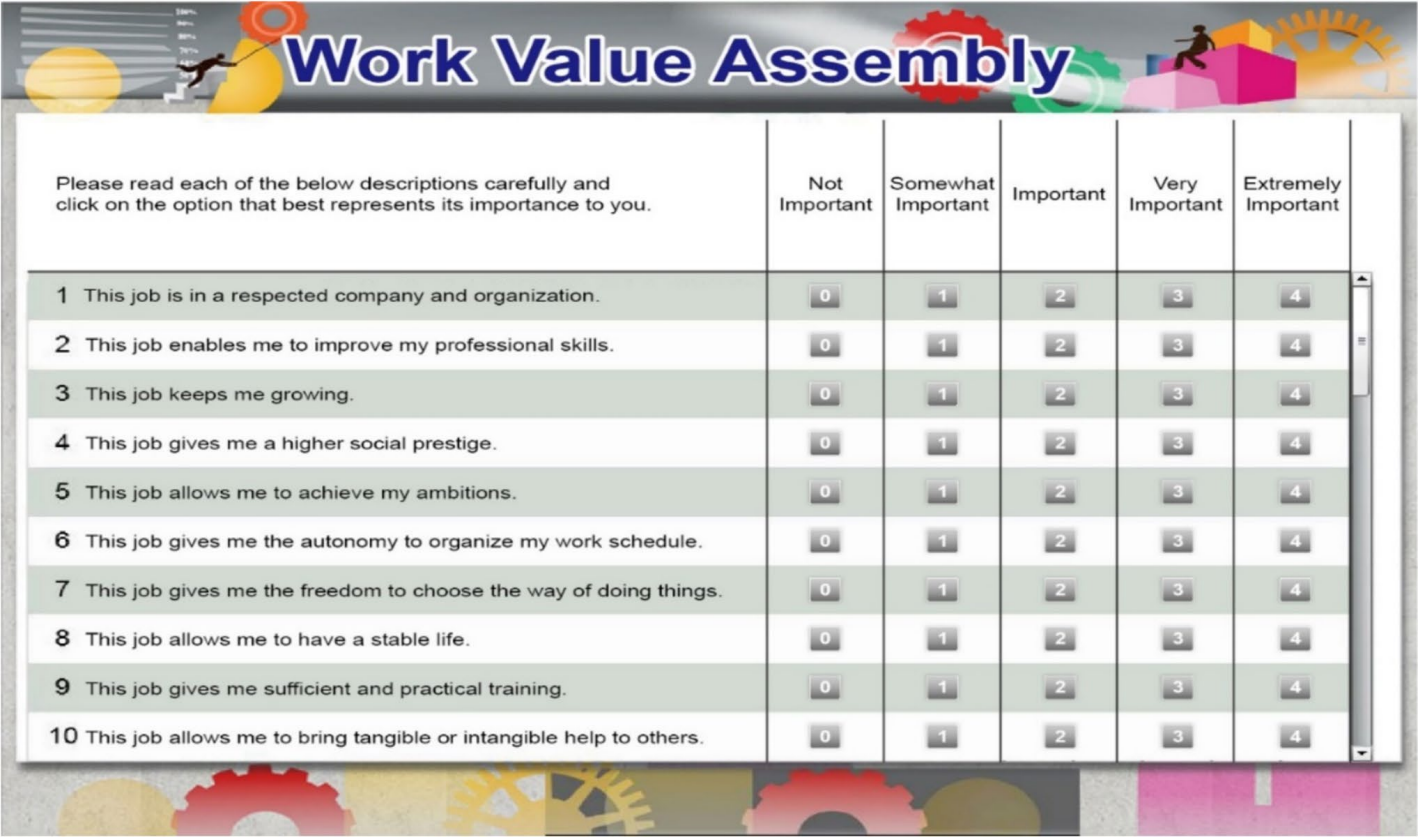
Screenshot of the Likert version of the Work Value Assembly (WVA) interface

The Likert-type version of the Work Values Assembly (WVA, Sung et al., 2019) was adopted to collect participants’ discrete item responses. The WVA consists of seven dimensions: prosocial motivation (PS), interpersonal connections (IP), prestige (PR), comfort (CS), professional growth (GR), self-actualization (SA), and autonomy (AU). Each dimension consists of six items, for a total of 42 items on the scale. Respondents rated the 42 items on the following five-point Likert-type scale: “not important,” “somewhat important,” “important,” “very important,” and “extremely important” (as shown in Fig. 3). Sung et al. (2019) indicated that the WVA has good reliability and validity in measuring the work values of Taiwanese high-school students.

#### Procedure

The test data were collected in March 2023. The purpose of the scale and the method of endorsing the item were explained to the participating students before the scale was administered to them. The administration of the WVA lasted for around 15–20 min.

#### Analysis

To verify the model-data fit of each WVA dimension, we utilized the CoRSA to analyze each dimension of the data independently. The outfit MNSQ values of each item in each dimension were computed using Eq. (21). In this study, the data were considered to fit the CoRSM adequately when the outfit MNSQ values of all retained items were at or below 1.4 (Bond & Fox, 2015; Wright & Linacre, 1994).

### Results

Table 8 indicates that the outfit MNSQ values for all items ranged from 0.48 to 0.86, so no items were excluded from the WVA. This result suggested that the participant’ responses to the items fit the model well. To verify the stability of the model-data fit, we split the full sample into two random subsamples and refitted the model separately to each subsample. The procedures were repeated ten times. In the first subsample, all items from the PS, IP, PR, CS, GR, SA, and AU dimensions of the WVA were identified as fitting in all ten replications. This result indicated that these items consistently fit the model. The second subsample produced similar results, confirming that all items were consistently identified as fitting across all dimensions.

**Table 8:** Results of the item parameter estimation for the Work Value Assembly (WVA)

Item	Overall difficulty estimate	*SE*	Outfit	Step difficulty estimate			
				Step 1	Step 2	Step 3	Step 4
PS1	0.10	0.05	0.48	− 3.03	− 1.01	1.01	3.03
PS2	0.08	0.05	0.59				
PS3	− 0.16	0.05	0.58				
PS4	0.10	0.05	0.65				
PS5	0.13	0.05	0.61				
PS6	− 0.25	0.05	0.55				
IP1	− 0.05	0.05	0.57	− 2.97	− 0.99	0.99	2.97
IP2	0.12	0.05	0.53				
IP3	0.18	0.05	0.61				
IP4	− 0.18	0.05	0.68				
IP5	− 0.05	0.05	0.62				
IP6	− 0.01	0.05	0.59				
PR1	0.38	0.04	0.61	− 2.25	− 0.75	0.75	2.25
PR2	0.31	0.04	0.61				
PR3	0.21	0.04	0.66				
PR4	− 0.52	0.04	0.67				
PR5	− 0.61	0.04	0.64				
PR6	0.23	0.04	0.73				
CS1	− 0.32	0.06	0.61	− 3.01	− 1.00	1.00	3.01
CS2	− 0.31	0.06	0.54				
CS3	0.10	0.06	0.70				
CS4	0.20	0.06	0.79				
CS5	− 0.09	0.06	0.58				
CS6	0.43	0.05	0.69				
GR1	− 0.07	0.05	0.67	− 3.31	− 1.10	1.10	3.31
GR2	− 0.34	0.05	0.53				
GR3	0.05	0.05	0.65				
GR4	− 0.60	0.06	0.66				
GR5	0.99	0.05	0.86				
GR6	− 0.03	0.05	0.63				
SA1	− 0.61	0.06	0.61	− 3.30	− 1.10	1.10	3.30
SA2	0.04	0.06	0.65				
SA3	0.01	0.06	0.63				
SA4	0.25	0.06	0.63				
SA5	0.14	0.06	0.72				
SA6	0.18	0.06	0.65				
AU1	0.13	0.05	0.59	− 3.52	− 1.17	1.17	3.52
AU2	0.34	0.05	0.61				
AU3	− 0.03	0.05	0.64				
AU4	− 0.15	0.05	0.58				
AU5	− 0.06	0.05	0.59				
AU6	− 0.23	0.05	0.68				

*Note*. PS = prosocial motivation; IP = interpersonal connections; PR = prestige; CS = comfort; GR = professional growth; SA = self-actualization; AU = autonomy

The SEs of all items ranged from 0.04 to 0.06, implying that the estimations of overall difficulty parameters were accurate. The four estimates of step difficulties for the PR dimension (– 2.25, – 0.75, 0.75, and 2.25) were more closely distributed than those for the AU dimension (– 3.52, – 1.17, 1.17, and 3.52). This indicated that respondents with low PR levels (e.g., – 3.00) had higher probabilities of choosing “not important,” and those with high levels (e.g., 3.00) had higher probabilities of choosing “extremely important.” In the AU dimension, respondents with low AU levels had higher probabilities of choosing “somewhat important” and those with high levels had higher probabilities of choosing “very important.”

Table 9 presents the means, SDs, and correlations among the seven measures in the ability estimates. The SA dimension had the highest mean estimate across the dimensions, which implied that respondents had a stronger preference for the job, enabling them to make use of their expertise. The PR dimension had the lowest mean estimate across the dimensions, which indicated that respondents had a weaker preference for the job enabling them to achieve a higher social status. The inter-trait correlation coefficient ranged from 0.23 to 0.65. The correlation was strongest between the GR and SA dimensions, which implied that when respondents had a stronger preference for gaining specialized skills while working at a job, they would also have a stronger preference for a job enabling them to make use of their expertise.

**Table 9:** Descriptive statistics and correlations of the person parameters for the Work Value Assembly (WVA)

Dimension	*M*	*SD*	PS	IP	PR	CS	GR	SA
PS	0.97	1.12	–					
IP	1.56	1.06	0.48	–				
PR	0.23	1.01	0.31	0.48	–			
CS	2.33	0.91	0.23	0.34	0.47	–		
GR	1.65	0.98	0.59	0.49	0.36	0.33	–	
SA	2.47	0.92	0.46	0.44	0.37	0.39	0.65	–
AU	1.54	1.07	0.33	0.42	0.45	0.40	0.50	0.60

*Note*. PS = prosocial motivation; IP = interpersonal connections; PR = prestige; CS = comfort; GR = professional growth; SA = self-actualization; AU = autonomy

In summary, the results of the above analyses demonstrated that the WVA items fit the CoRSM well. Based on the well model-data fit, the CoRSA is capable of estimating work value scores in each dimension for each individual. These estimates meet the requirements for applying parametric statistics to interval scores (Bond & Fox, 2015; Harwell & Gatti, 2001; Iramaneerat et al., 2008; Wright & Mok, 2004), thereby facilitating more precise and reliable measurement of work values.

#### Summary of Study 3 A and 3B

We applied the CoRSA to both a continuous dataset and a discrete dataset, demonstrating how to assess the degree of fit between the empirical data and the model using item fit statistics. This process is crucial for validating the benefits of the Rasch family models, including the CoRSM. By verifying that the empirical data fit the CoRSM, CoRSA effectively transforms both continuous and discrete raw data into interval-level scores. This transformation is critical because it allows researchers and practitioners to have confidence that the data meet the requirements necessary for conducting parametric statistical analyses (Embretson, 1996; Harwell & Gatti, 2001; Iramaneerat et al., 2008; Wright & Mok, 2004).

## Study 4: The implementation of the Continuous Rating Scale Analytics (CoRSA)

To assist researchers and practitioners in analyzing their own continuous and discrete data collected from rating scales, the fourth study aimed to integrate CoRSA into the VAS-RRP 2.0 platform (Sung & Wu, 2018; http://vasrrp.net/vasrrp2). Below, we explain how to implement CoRSA to rescale continuous or discrete data to interval scores.

### Step 1: Designate the data properties of scale scores

**Fig. 4:**
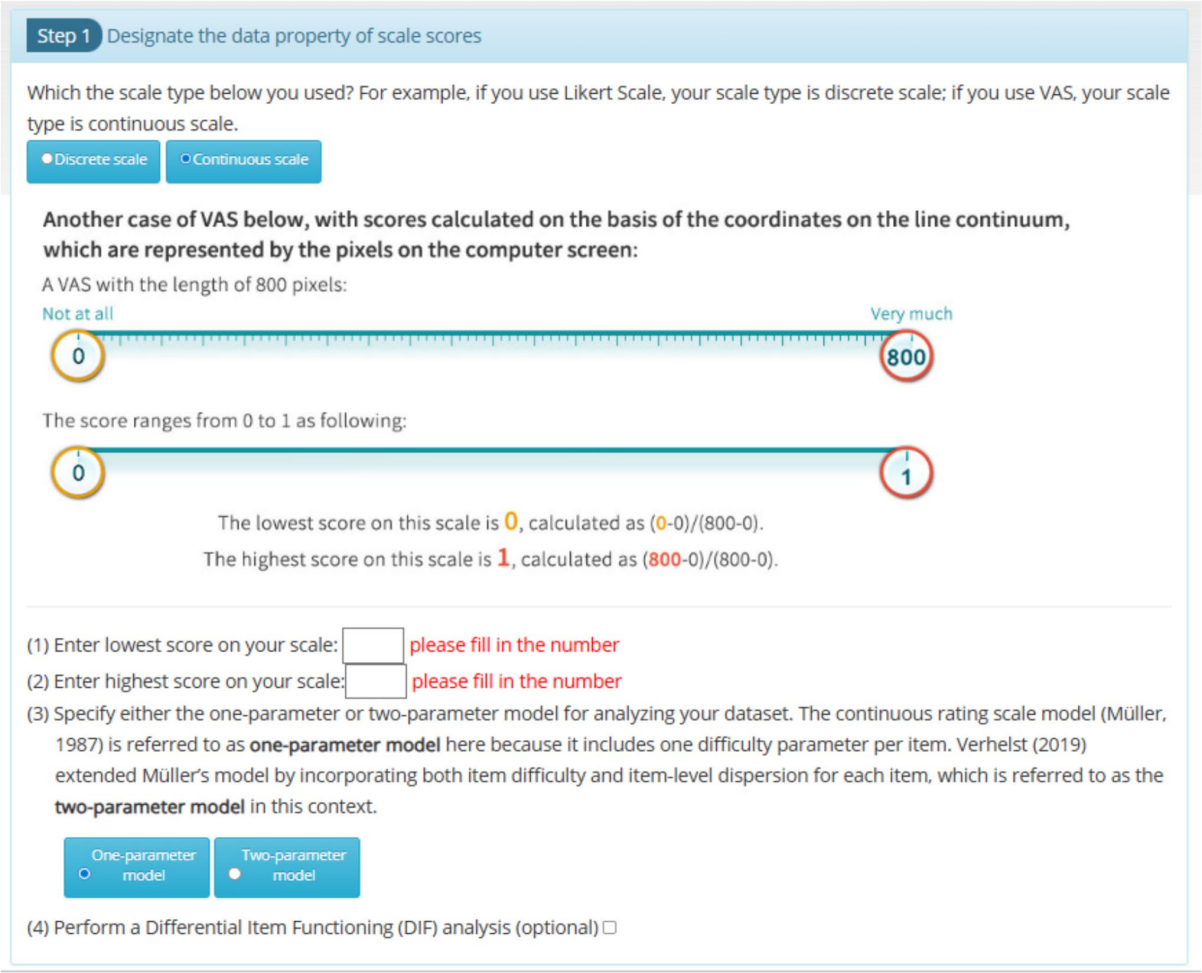
Designating the data properties of scale scores

Users must specify whether their dataset contains discrete or continuous scale scores and define the original score range of the scale used during data collection. For example, scores may originate from a five-point Likert scale or range from 0 to 800 (see Fig. 4). Additionally, users can choose to analyze continuous datasets using either Müller’s model or Verhelst's model.

### Step 2: Download the template for your data file

**Fig. 5:**
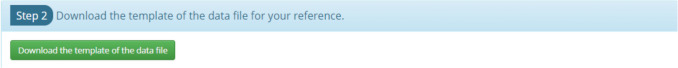
Downloading the template of the data file for your reference

CoRSA provides a template file formatted according to its required data structure (see Fig. 5). Users must submit their response data in this specified format to ensure that CoRSA can correctly process information about participants and item features.

### Step 3: Upload the data file and obtain the result file

**Fig. 6:**
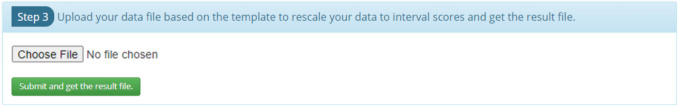
Submitting and getting the result file

To perform the analysis, users should upload their completed data file by clicking the "Choose File" button and selecting the appropriate file. Then, click the "Submit and get the result file" button to start the estimation process. Upon completion, the system will generate and download a result file containing interval estimates of the ability, difficulty, and dispersion parameters (see Fig. 6).

**Fig. 7:**
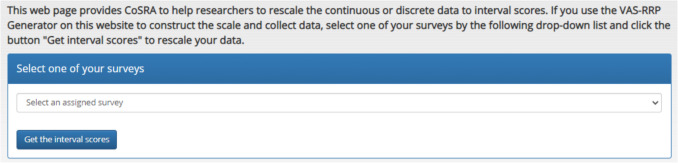
Using the CoRSA to analyze the response data collected by the VAS-RRP generator

It is noteworthy that users who are interested in constructing a scale and collecting data using the VAS-RRP Generator may skip steps 1–3 and instead simply select their surveys and click the “Get interval scores” button to rescale their raw data (see Fig. 7). The functions and procedure of the VAS-RRP Generator have been described by Sung and Wu (2018).

**Fig. 8:**
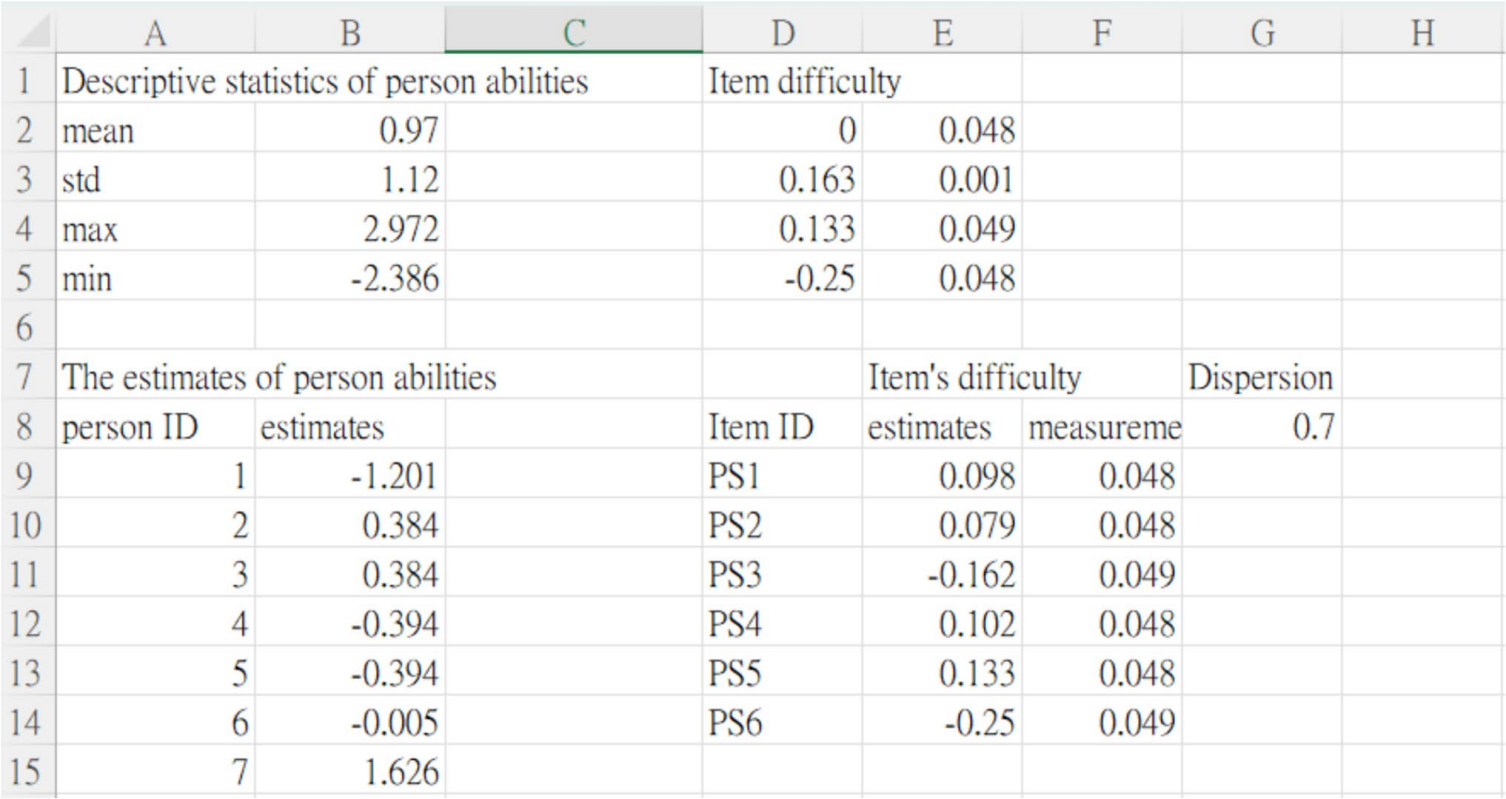
Screenshot of the CoRSA output file

**Fig. 9:**
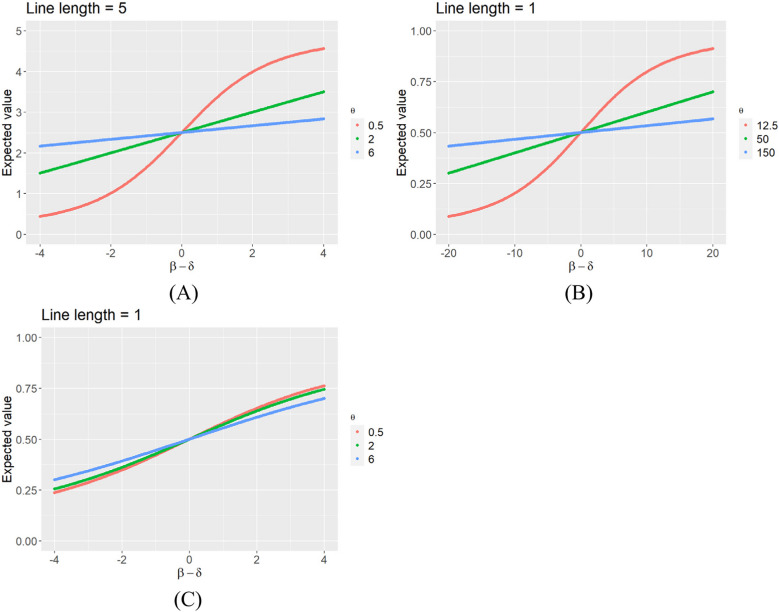
Item characteristic curves for three θ values. *Note.* Expected item responses for different values of the dispersion parameter and line length: (**A**) *L* = 5 and *θ* = 0.5, 2, and 6; (**B**) *L* = 1 and *θ* = 12.5, 50, and 150; and (**C**) *L* = 1 and *θ* = 0.5, 2, and 6

The results of the analysis were systematically organized and stored in a worksheet within a Microsoft Excel file. For example, the upper part of the worksheet displayed descriptive statistics for the ability estimates of the entire sample, along with the difficulty estimates for all items (see Fig. 8). Additionally, the worksheet included the individual estimates of person abilities, item difficulties, and a test-level dispersion parameter, providing a comprehensive summary of the parameter estimation results.

For readers who require more detailed instructions on conducting CoRSM analysis, or who wish to perform differential item functioning (DIF) analysis to evaluate the measurement fairness of rating scales using CoRSA, a worked example is available at: https://osf.io/c3jzy/?view_only=0314d5f0cbef4627812ac0bf5d54d271.

## Discussion

Advances in computer technology have addressed practical challenges in collecting and scoring continuous data, facilitating the use of VASs (Reips & Funke, 2008). Within the framework of IRT, several models have been proposed to use the VAS-typed continuous responses as indicators of person-level abilities and to model an examinee’s expected rating as a function of person ability and item difficulty parameters. Samejima (1973) developed the CRM by treating continuous responses as a limiting case of graded responses, where the number of categories is infinitely large. Samejima’s study also explored open and closed response situations, inspiring subsequent development of models for continuous data. To manage more complex data structures, Noel and Dauvier (2007) proposed the BRM to address complexities of response distributions such as asymmetry of bounded continuous data, by assuming the item response follows a beta distribution. Recent advancements have led to the creation of accessible tools like the EstCRM (Zopluoglu, 2012) and the sirt (Robitzsch, 2024) R packages, designed to conduct parameter estimation for CRM and BRM. These developments have broadened the applicability of IRT modeling to continuous data. Nevertheless, several challenges persist in employing these models and tools for continuous data analysis. Primarily, the CRM does not address the issue of measurement scale levels, raising concerns about its capability to produce interval-level estimates for person and item parameters—crucial for conducting parametric statistical analyses (Harwell & Gatti, 2001; Iramaneerat et al., 2008; Wright & Mok, 2004). Furthermore, the mathematical complexity underlying the BRM and the implementation of the sirt R package may be difficult for general users in the social sciences to master, potentially limiting its use and popularity.

Among the various continuous IRT models, the CoRSM developed by Müller (1987) offers several advantageous features for practical applications. Firstly, it allows examinees to mark any point along a continuum, which eliminates the need for data transformations that prevent scores at scale boundaries (Molenaar et al., 2022). Secondly, the CoRSM is an extension of the rating scale model (Andrich, 1978), enabling it to accommodate not only continuous responses (e.g., markings on a VAS) but also discrete responses (e.g., ratings on a Likert-type scale). Most importantly, as a member of the Rasch model family (Noel & Dauvier, 2007), the CoRSM theoretically provides interval-level estimates for both persons and items, which are crucial for conducting parametric statistical procedures (Harwell & Gatti, 2001; Iramaneerat et al., 2008; Wright & Mok, 2004). However, the widespread adoption of the CoRSM may be hindered by the lack of efficient algorithms and validated analytical tools for parameter estimation. To date, the pcIRT R package (Hohensinn, 2018) appears to be the only available tool for estimating item and person parameters for the CoRSM. Nevertheless, the use of pcIRT comes with significant limitations. Firstly, the parameter recovery of pcIRT has not been thoroughly validated, which casts doubt on its accuracy for estimating person and item parameters for the CoRSM. Secondly, pcIRT does not address the issue of line length being related to the unit of the latent continuum. Consequently, pcIRT struggles to obtain accurate estimates because it uses algorithms that rescale the observed scale scores to a specified interval without modifying the unit of the latent trait scale. Additionally, implementing pcIRT requires users to engage with R programming, making it challenging for those unfamiliar with the R environment, which could limit its utility and popularity.

In this article, we conducted four studies to address the technical challenges associated with estimating parameters of the CoRSM. These studies provide more convincing evidence for the effectiveness of using the algorithms of continuous scores in both simulation and empirical data. Additionally, we offer a user-friendly interface for researchers and practitioners interested in collecting and analyzing continuous data. The first study proposed the Al-CoRSM algorithm, which utilized MML and MAP methods to estimate person and item parameters of the CoRSM. This study also developed the analytical tool CoRSA by implementing Al-CoRSM for researchers and practitioners to conduct parameter estimation. CoRSA boasts several notable advantages. Firstly, simulation studies show that CoRSA achieves effective parameter recovery across various testing conditions. It is observed that increasing the sample size leads to reduced estimation errors for item difficulties, while longer tests decrease estimation error for person abilities. These results align with previous research findings (Noel & Dauvier, 2007; Wang & Zeng, 1998). Furthermore, it is also noted that increasing dispersion parameters reduces the accuracy of estimations for both person abilities and item difficulties. Moreover, the MML and MAP methods implemented by CoRSA exhibit robustness, as evidenced by similar estimation accuracy for non-normal ability distributions compared to normal distributions.

The second study examined the capability of CoRSA to analyze continuous and discrete data. We verified the second key advantage of the CoRSA algorithm, which is its ability to adjust the unit of the trait scale when rescaling observed data to a specified interval, thus maintaining constant response probabilities. This critical feature, absent in pcIRT, allows for more accurate parameter estimation, especially when observed data are rescaled to the unit interval. This capability is particularly crucial in practical applications where data transformation is routinely required. Simulation studies have shown that rescaling data in pcIRT without adjusting the unit of the latent scale leads to parameter estimates that significantly diverge from the true values. In contrast, CoRSA is adept at providing satisfactory parameter estimates under these conditions, making it a more reliable tool for practical applications. These findings provide compelling evidence to support the superior performance of CoRSA. Furthermore, the capability of CoRSA to analyze discrete data was also verified. Given that continuous response formats can be viewed as an extension of graded response formats (Mellenbergh, 1994; Samejima, 1973), it theoretically suggests that the CoRSM is applicable to discrete data, such as those collected from Likert-type scales. Simulation studies tested the parameter recovery of CoRSA on discrete data with five, seven, and nine categories, and the results have shown that its performance is very comparable to that of ConQuest, a well-known software in IRT modeling. This finding not only supports the theoretical postulate that the CoRSM can effectively handle discrete data but also highlights the utility of CoRSA in providing accurate parameter estimates for such data. This versatility is particularly beneficial for researchers who deal with both continuous and discrete data formats, allowing them to utilize a single, robust methodological approach for diverse data types.

In the third study, we verified CoRSA’s capability to transform continuous raw data into interval scores. This transformation ensures that both person abilities and item difficulties estimated by CoRSA conform to an interval scale of measurement, provided that the data fit the CoRSM. This feature is particularly valuable as it aligns with the standards required for applying parametric statistical procedures. For discrete data, the benefits of transforming ordinal discrete responses into interval Rasch scores are well documented, yet these benefits are contingent on the data fitting the requirements of the Rasch family models (Bond & Fox, 2015; Wright & Mok, 2004). Given that the CoRSM is an extension of the RSM (Andrich, 1978), it inherits similar properties. Therefore, when the data fit the CoRSM, the ordinal continuous item responses can be rescaled using CoRSA to produce interval estimates for persons and items. This rescaling meets the requirements for conducting rigorous parametric statistics (Dumenci & Achenbach, 2008; Embretson, 1996; Harwell & Gatti, 2001; Iramaneerat et al., 2008; Li, 2016; Wright & Mok, 2004). The rescaling of scores from ordinal to interval levels is critical because using parametric statistics (e.g., *t* tests and ANOVA) on ordinal scores can lead to several statistical issues. CoRSA addresses these issues by enabling the application of parametric statistical procedures to interval-level scores derived from continuous scales, thereby avoiding the drawbacks associated with ordinal data analysis.

The fourth study addressed the issue of enhancing the accessibility and usability of psychometric tools for analyzing continuous data, which has long been a challenge for researchers who may not be familiar with the detailed theories and methods of psychometric measurement. The fourth study integrated CoRSA into the VAS-RRP 2.0 website, significantly simplifying the process for researchers to collect and rescale their continuous and discrete data sets to interval scores. This integration into a user-friendly web-based platform is poised to have a substantial impact on the field of educational and psychological assessment. By streamlining the rescaling process, CoRSA enables researchers, regardless of their background in psychometrics, to achieve more precise and accurate measurements. The availability of CoRSA on a platform like VAS-RRP 2.0 allows a broader range of professionals to employ advanced psychometric analyses in their work, and it fills the gap of lacking valid tools for analyzing continuous data compared to the abundance of tools for analyzing discrete data.

The CoRSA estimates item parameters using the MML method proposed by Bock and Aitkin (1981), which offers several advantages. It provides consistent item parameter estimates and is applicable across various IRT models (Baker & Kim, 2004; Bock & Aitkin, 1981). However, an important limitation of the MML framework lies in its reliance on an assumed latent trait distribution—typically the standard normal distribution. If this distributional assumption is violated, parameter estimates may become biased. Nonetheless, Embretson and Reise (2000) indicated that MML-based estimates are generally robust to moderate deviations from normality. The findings from Study 2A corroborate this, showing that even when the true ability distribution was non-normal, the CoRSA still yielded accurate item parameter estimates, as reflected in the MAD and RMSE values. In addition, it is notable to acknowledge that the computational burden of MML increases exponentially with the number of latent dimensions in multidimensional models. This is due to the need for multidimensional integration over the latent trait distribution (Cui et al., 2024; Rijmen, 2009). Future development of the CoRSA may therefore consider incorporating alternative estimation strategies to address issues in high-dimensional applications.

The present findings indicate several implications for future research, particularly regarding the scope and capabilities of the analytical tool CoRSA developed in this study. In addition to demonstrating the validity and efficacy of Al-CoRSM and CoRSA, this research also provides researchers/practitioners with persuasive examples of using continuous scales to collect data on psychological traits. Researchers/practitioners who are interested in issues such as comparing the measurement errors of scales with various levels of coarseness, investigating the intervalness of continuous scales, and exploring the possibility of creating partially ipsative scales through continuous scales would be empowered to obtain more solid evidence by using valid and easily accessible tools such as CoRSA. Furthermore, based on the CoRSM, CoRSA currently supports only unidimensional analysis of continuous data. This restriction becomes particularly limiting when analyzing multidimensional data, such as when assessing multiple psychological traits simultaneously. In cases where test data are multidimensional, it is necessary to perform analyses on each dimension independently. This approach not only increases the time required for analysis as the number of dimensions grows, but also fails to consider the intercorrelations among these dimensions. Consequently, this method allows only partial extraction of information from the test data, potentially leading to less accurate estimations of the subjects' latent traits. To address these limitations, future research should focus on the development of multidimensional models for continuous data. These models would enable the estimation of person abilities and item difficulties across multiple dimensions in a single analytical framework. By accounting for the correlations between dimensions, such models would enhance the capability of CoRSA to extract more comprehensive information regarding the exact levels of the subjects’ latent traits. This would not only improve the accuracy of the measurements but also reduce measurement errors, thereby providing a more accurate assessment of the latent traits.

## Electronic supplementary material

Below is the link to the electronic supplementary material.

Supplementary file1 (ZIP 1608 KB)

## Data Availability

The datasets analyzed in the third study are available in the Open Science Framework repository at https://osf.io/c3jzy/?view_only=0314d5f0cbef4627812ac0bf5d54d271.

## Appendix 1 Item characteristic curves for the continuous rating scale model

Subplot A of Fig. 9 presents the item characteristic curves for three *θ* values when item response *x* is distributed within interval [0, 5] (which is equivalent to a line length of 5). Because the item characteristic curve is a monotonic function, a larger value of $\beta-\delta$ corresponds to a larger expected value, indicating a higher probability that an examinee will mark a point closer to the right end of the horizontal line (or closer to the top of the vertical line), which implies that the examinee has a stronger preference for a certain option. The parameter *θ* reflects the shape of the rating distribution (Andrich, 1982): *θ* = 0.5 corresponds to the item characteristic curve having lower and upper asymptotes of 0.5 and 4.5, respectively, indicating that examinees’ item responses are distributed over a wide interval; while *θ* = 6.0 corresponds to lower and upper asymptotes of 2.2 and 2.8, respectively, depicting that item responses are expected to be distributed within a narrow interval. It is clear that when the value of $\beta-\delta$ is kept constant, a smaller *θ* indicates a larger variance of the item responses, which leads to higher discrimination (Verhelst, 2019). The item characteristic curves for line lengths of 5 and 1 are shown in subplots A and B of Fig.9, respectively; $\beta$ and $\delta$ were multiplied by 5 while *θ* was multiplied by 25 in subplot B to keep the probability densities of the item responses unchanged and thus maintain the shape of the response distributions. Obviously, the line length relates to the unit of measurement used for the latent trait variable (Müller, 1987). In other words, rescaling observed item responses *x* to any specified interval is equivalent to changing the line length, and thus changing the unit of the latent trait scale. If the observed item responses are rescaled while keeping the values of $\beta$, $\delta$, and *θ* constant, this will change the shape of the response distributions, as shown in subplot C of Fig. 9. This issue is discussed in more detail in Study 2A.

### Indeterminacy of parameter estimates

In addition to the estimation methods, another issue in IRT modeling is the indeterminacy of parameter estimates, referring to the origin and unit of the latent continuum (i.e., measurement on the latent trait scale) being nonunique due to multiple values of person and item parameters resulting in the same response probability (de Ayala, 2009). For the purpose of illustrating the indeterminacy problem for the CoRSM, and in accordance with the example shown by Humphry (2008) for the Rasch model, we add two constants *k* and $\rho$ to Eq. (1) to yield the probability density of *x* as

22 $$f\left(X=x\vert\beta,\delta,\theta\right)=\frac{exp\left[x\rho\left(\beta^\ast-\delta^\ast\right)+x\rho\left(2c-x\right)\theta^{ast}\right]}{\int_{c-L/2}^{c+L/2}exp\left[t\rho\left(\beta^\ast-\delta^\ast\right)+t\rho\left(2c-t\right)\theta^\ast\right]dt}$$

where ${\beta}^{\ast}=\left(\beta+k\right)/\rho$, ${\delta}^{\ast}=\left(\delta+k\right)/\rho$, and ${\theta }^{\ast}=\theta /\rho$. To preserve simplicity without loss of generality, the person and item subscripts are temporally omitted in this section. Equation 22 is equivalent to Eq. (1) with *k* and $\rho$ taking values of 0 and 1, respectively. Because constant *k* cancels out when computing the difference between $\beta$ and $\delta$, there is no change in the probability density between Eqs. (1) and 22. The origin of the latent continuum is nonunique because constant *k* can be arbitrarily chosen to be any value. Similarly, even if *k* = 0, the unit of the continuum is indeterminate because constant $\rho$ can be specified to be any positive value without changing the probability density of Eq. 22. Furthermore, if $\rho \ne 1$, then in general ${\beta }^{\ast}\ne \beta$, ${\delta}^{\ast}\ne \delta$, and ${\theta}^{\ast}\ne \theta$. It is therefore obvious that changing the value of $\rho$ changes the unit of the continuum.

In addition to constants *k* and $\rho$, Müller (1987) indicated that the line length relates to the unit of the latent continuum. Verhelst (2019) demonstrated that rescaling the observed data changes the unit of the latent continuum. Suppose the VAS is represented by a line segment of length *L*, and the lower and upper bounds of random variable* Y* are defined as 0 and *L*, respectively. Because the variable can be arbitrarily rescaled to any specified interval, which leads to the line length changing, this also changes the bounds of the integral. If variable *Y* is rescaled to the unit interval, which leads to a new variable $U=Y/L$, and the lower and upper bounds change to 0 and 1, respectively. The probability density of variable *U* is given by

23 $$\begin{array}{lc}f\left(U=u\vert\beta \vert\delta \vert\theta \right)=\frac{exp\left[y\left(\beta -\delta \right)+y\left(L-y\right)\theta \right]}{\int\nolimits _{0}^{L}exp\left[t\left(\beta -\delta \right)+t\left(L-t\right)\theta \right]dt}\\ \ \ \ \ \ \ \ \ \ \ \ \ \ \ \ \ \ \ \ \ =\frac{exp\left[uL\left(\beta -\delta \right)+uL\left(L-uL\right)\theta \right]}{L{\int }_{0}^{1}exp\left[t\left(\beta -\delta \right)+t\left(L-t\right)\theta \right]dt}\\ \ \ \ \ \ \ \ \ \ \ \ \ \ \ \ \ \ \ \ \ =\frac{1}{L}f\left(U=u\vert{\beta}^{^\prime}|{\delta}^{^\prime}\vert{\theta}^{^\prime}\right)\end{array}$$

where ${\beta}^{^\prime}=L\beta$, ${\delta}^{^\prime}=L\delta$, and ${\theta}^{^\prime}={L}^{2}\theta$. An instance of this derivation is that halving the response variable (i.e., $U=Y/2$) will double the values of the person estimates, and thus double the mean and standard deviation (SD) of the ability distribution. It is therefore clear that rescaling the observed variable changes the bounds of the integration, and thus affects the unit of the latent continuum (Verhelst, 2019).

An implication of these viewpoints is that estimation algorithms and analytical tools should accommodate practical requirements when rescaling data, and take the changes in the line length into account to modify the unit of the latent scale when performing parameter estimation. The consequences of rescaling the response variable without modifying the unit of the scale were investigated in this Study 2A.

## Appendix 2: Detailed derivations for the Al-CoRSM

### Derivatives of the log-likelihood function with respect to item difficulty parameter

#### First derivatives of the log-likelihood function

24 $$\begin{array}{lc}\frac{\partial \mathrm{ln}(L)}{\partial {\delta}_{i}}=\sum\limits_{n=1}^{N}\frac{1}{P({X}_{n})}\frac{\partial P({X}_{n})}{\partial {\delta }_{i}}\\ \ \ \ \ \ \ \ =\sum\limits_{n=1}^{N}\frac{1}{P({X}_{n})}\int\nolimits _{-\infty }^{+\infty}\frac{\partial f\left({x}_{ni}\vert\Theta \right)}{\partial {\delta}_{i}}\left(\prod\limits_{k=1,k\ne i}^{I}f\left({x}_{nk}|\Theta \right)\right)g(\beta)d\beta \\ \ \ \ \ \ \ \ =\sum\limits_{n=1}^{N}\frac{1}{P({X}_{n})}\int\nolimits _{-\infty }^{+\infty }\frac{\partial f\left({x}_{ni}\vert\Theta \right)}{\partial {\delta}_{i}}\frac{P({X}_{n}\vert\Theta)}{f\left({x}_{ni}\vert\Theta \right)}g(\beta)d\beta \end{array}$$

Equation 24 can be approximated using the Gauss–Hermite quadrature as follows:

25 $$\begin{array}{lc}\frac{\partial \mathrm{ln}(L)}{\partial {\delta}_{i}}= \sum\limits_{n=1}^{N}\frac{1}{P({X}_{n})}\sum\limits_{q= \mathrm{1} }^{Q}\left(P({X}_{n}\vert{V}_{g},{\delta}_{i},\theta)\frac{\partial f({x}_{ni}\vert{V}_{g},{\delta}_{i},\theta)}{\partial {\delta}_{i}}\frac{1}{f({x}_{ni}\vert{V}_{g},{\delta}_{i},\theta)}\right)\\ \ \ \ \ \ \ \ =\sum\limits_{n=1}^{N}\frac{1}{P({X}_{n})}\sum\limits_{q= \mathrm{1} }^{Q}\left({L}_{n}({V}_{g})A({V}_{g})\frac{\partial f({x}_{ni}\vert{V}_{g},{\delta}_{i},\theta)}{\partial {\delta}_{i}}\frac{1}{f({x}_{ni}|{V}_{g},{\delta}_{i},\theta)}\right)\\ \ \ \ \ \ \ \ =\sum\limits_{n=1}^{N}\sum\limits_{q= \mathrm{1} }^{Q}\left(\frac{{L}_{n}({V}_{g})A({V}_{g})}{\sum\nolimits _{q= \mathrm{1} }^{\mathrm{Q}}{L}_{n}({V}_{g})A({V}_{g})}\frac{\partial f({x}_{ni}|{V}_{g},{\delta}_{i},\theta)}{\partial {\delta}_{i}}\frac{1}{f({x}_{ni}\vert{V}_{g},{\delta}_{i},\theta)}\right)\end{array}$$

with

26 $${L}_{n}\left({V}_{q}\right)=\prod\limits_{i=1}^{I}f\left({x}_{ni}|{V}_{q}|{\delta }_{i}|\theta \right)$$

where *V*_*q*_ is a quadrature point and *A*(*V*_*q*_) is the weight of the Gauss–Hermite quadrature, which is approximately the standard normal probability density at *V*_*q*_, and $\sum\limits_{q=1}^{Q}A\left({V}_{q}\right)=1$. *Q* is the total number of quadrature points. ${L}_{n}\left({V}_{q}\right)$ is the conditional probability of response vector ${\mathbf{X}}_{n}$ at ${V}_{q}$.

Let $$a=exp\left[-\theta {\left({x}_{ni}-\frac{{V}_{q}-{\delta}_{i}}{2\theta}\right)}^{2}\right]$$, and $$\gamma =\int\nolimits _{-d/2}^{d/2}exp\left[-\theta {\left(t-\frac{{V}_{q}-{\delta }_{i}}{2\theta}\right)}^{2}\right]dt$$

then

27 $$\frac{\partial f\left({x}_{ni}|{V}_{q}\vert{\delta}_{i}|\theta \right)}{\partial {\delta}_{i}}=\frac{\partial}{\partial {\delta}_{i}}\left[\frac{a}{r}\right]=\frac{{a}_{\delta}^{^\prime}\gamma-a{\gamma}_{\delta}^{^\prime}}{{\gamma}^{2}}$$

where

28 $$\begin{array}{lc}{a}_{\delta}^{^\prime}=\frac{\partial}{\partial {\delta}_{i}}exp\left[-\theta {\left({x}_{ni}-\frac{{V}_{f}-{\delta}_{i}}{2\theta}\right)}^{2}\right]=exp\left[-\theta {\left({x}_{ni}-\frac{{V}_{f}-{\delta}_{i}}{2\theta}\right)}^{2}\right]\left(-{x}_{ni}+\frac{{V}_{f}-{\delta}_{i}}{2\theta}\right)\\ {\gamma}_{\delta}^{^\prime}=\frac{\partial \gamma}{\partial {\delta}_{i}}={\int\limits_{-d/2}^{d/2}}exp\left[-\theta {\left(t-\frac{{V}_{f}-{\delta }_{i}}{2\theta }\right)}^{2}\right]\left(-t+\frac{{V}_{f}-{\delta }_{i}}{2\theta }\right)dt\end{array}$$

#### Second derivatives of the log-likelihood function

29 $$\frac{{\partial}^{2}ln\left(L\right)}{\partial {\delta}_{i}^{2}}=\sum\limits_{n=1}^{N}\frac{\partial}{\partial {\delta}_{i}}\left[\frac{1}{P\left({\mathbf{X}}_{n}\right)}\frac{\partial P\left({\mathbf{X}}_{n}\right)}{\partial {\delta}_{i}}\right]=\sum\limits_{n=1}^{N}\frac{1}{P{\left({\mathbf{X}}_{n}\right)}^{2}}\left[\frac{{\partial}^{2}P\left({\mathbf{X}}_{n}\right)}{\partial {\delta}_{i}^{2}}P\left({\mathbf{X}}_{n}\right)-{\left(\frac{\partial P\left({\mathbf{X}}_{n}\right)}{\partial {\delta }_{i}}\right)}^{2}\right]$$

30 $$\frac{{\partial}^{2}ln\left(L\right)}{\partial {\delta}_{i}\partial {\delta }_{j}}=\sum\limits_{n=1}^{N}\frac{1}{P{\left({\mathbf{X}}_{n}\right)}^{2}}\left(\frac{{\partial }^{2}P\left({\mathbf{X}}_{n}\right)}{\partial {\delta}_{i}\partial {\delta}_{j}}P\left({\mathbf{X}}_{n}\right)-\frac{\partial P\left({\mathbf{X}}_{n}\right)}{\partial {\delta}_{i}}\frac{\partial P\left({\mathbf{X}}_{n}\right)}{\partial {\delta}_{j}}\right)$$

where

31 $$\begin{array}{lc}\frac{\partial P\left({\mathbf{X}}_{n}\right)}{\partial {\delta}_{i}}=\int\nolimits_{-\infty}^{+\infty}\frac{\partial f\left({x}_{ni}|\Theta \right)}{\partial {\delta}_{i}}\left(\prod\limits_{k=1,k\ne i}^{\mathrm{I}}f\left({x}_{nk}\vert\Theta \right)\right)g\left(\beta \right)d\beta \\ \ \ \ \ \ \ \ \ \ =\int\nolimits _{-\infty }^{+\infty }\frac{\partial f\left({x}_{ni}|\Theta \right)}{\partial {\delta}_{i}}\frac{P\left({\mathbf{X}}_{n}\vert\Theta \right)}{f\left({x}_{ni}\vert\Theta \right)}g\left(\beta \right)d\beta\end{array}$$

32 $$\begin{array}{lc}\frac{{\partial}^{2}P\left({\mathbf{X}}_{n}\right)}{\partial {\delta}_{i}^{2}}=\frac{\partial}{\partial {\delta}_{i}}\int\nolimits _{-\infty}^{+\infty}\frac{\partial f\left({x}_{ni}|\Theta \right)}{\partial {\delta}_{i}}\left(\prod\limits_{k=1,k\ne i}^{\mathrm{I}}f\left({x}_{nk}|\Theta \right)\right)g\left(\beta \right)d\beta \\ \ \ \ \ \ \ \ \ \ \ =\int\nolimits _{-\infty}^{+\infty}\frac{{\partial}^{2}f\left({x}_{ni}|\Theta \right)}{\partial {\delta}_{i}^{2}}\frac{P\left({\mathbf{X}}_{n}\vert\Theta \right)}{f\left({x}_{ni}|\Theta \right)}g\left(\beta \right)d\beta\end{array}$$

33 $$\frac{{\partial}^{2}f\left({x}_{ni}\vert{V}_{q}|{\delta}_{i}\vert\theta \right)}{\partial {\delta}_{i}^{2}}=\frac{\partial}{\partial {\delta}_{i}}\left(\frac{{a}_{\delta}^{^\prime}\gamma-a{\gamma}_{\delta}^{^\prime}}{{\gamma}^{2}}\right)=\frac{{a}_{\delta}^{^{\prime\prime} }{\gamma}^{2}-a\gamma {\gamma}_{\delta }^{^{\prime\prime} }-2{a}_{\delta}{\prime}\gamma {\gamma}_{\delta}^{^\prime}+2a{{\gamma}^{^\prime}}_{\delta}^{2}}{{\gamma}^{3}}$$

34 $${a}_{\delta}^{^{\prime\prime}}=\frac{\partial {a}_{\delta}^{\prime}}{\partial {\delta}_{i}}=exp\left[-\theta {\left({x}_{ni}-\frac{{V}_{q}-{\delta}_{i}}{2\theta}\right)}^{2}\right]\left[{\left(-{x}_{ni}+\frac{{V}_{q}-{\delta}_{i}}{2\theta }\right)}^{2}-\frac{1}{2\theta }\right]$$

35 $${\gamma}_{\delta}^{^{\prime\prime} }=\frac{\partial {\gamma}_{\delta}^{^\prime}}{\partial {\delta}_{i}}=\int\nolimits _{-d/2}^{d/2}exp\left[-\theta {\left(t-\frac{{V}_{q}-{\delta}_{i}}{2\theta }\right)}^{2}\right]\left[{\left(-t+\frac{{V}_{q}-{\delta }_{i}}{2\theta }\right)}^{2}-\frac{1}{2\theta }\right]dt$$

### Derivatives of the log-likelihood function with respect to person parameter

#### First derivatives of the log-likelihood function

Suppose that examinees are randomly sampled from a population where the latent trait is distributed according to a density function $g\left(\beta\right)$ as follows:

36 $$\mathrm{g}\left(\beta\right)={\left(2{\pi\sigma}^{2}\right)}^{-\frac{1}{2}}exp\left[-\frac{1}{2}{\left(\frac{\beta-\mu}{\sigma}\right)}^{2}\right]$$

The first derivative of the log posterior function is

37 $$\begin{array}{l}\frac{\partial ln{f}^{\ast}\left({\beta}_{n}|{\mathbf{X}}_{n}\right)}{\partial {\beta}_{n}}=\frac{\partial ln\left[P\left({\mathbf{X}}_{n}|{\beta}_{n}\right)\right]}{\partial {\beta}_{n}}+\frac{\partial ln\left[g\left({\beta}_{n}\right)\right]}{\partial {\beta}_{n}}\\ \ \ \ \ \ \ \ \ \ \ \ \ \ \ =\sum\limits_{\mathrm{i}=1}^{\mathrm{I}}\frac{\partial}{\partial {\beta}_{n}}lnf\left({x}_{ni}|\Theta \right)-\left(\frac{{\beta}_{n}-\mu}{{\sigma }^{2}}\right)\end{array}$$

where $\mu$ and ${\sigma}^{2}$ are the mean and variance of the $g\left(\beta \right)$.

Let $$A=-\theta {\left({x}_{ni}-\frac{{\beta}_{n}-{\delta}_{i}}{2\theta}\right)}^{2}$$, then

38 $$\frac{\partial}{\partial {\beta}_{n}}lnf\left({x}_{ni}|\Theta \right)=\frac{\partial}{\partial {\beta}_{n}}\left[ln\left({e}^{A}\right)-ln\gamma \right]={A}_{\beta}^{^\prime}-\frac{{\gamma}_{\beta}^{\prime}}{\gamma}$$

where

39 $${A}_{\beta}^{^\prime}=\frac{\partial }{\partial {\beta }_{n}}\left[-\theta {\left({x}_{ni}-\frac{{\beta}_{n}-{\delta}_{i}}{2\theta}\right)}^{2}\right]=-\left(-{x}_{ni}+\frac{{\beta}_{n}-{\delta}_{i}}{2\theta}\right)$$

40 $${\gamma}_{\beta}^{\prime}=\frac{\partial \gamma}{\partial {\beta}_{n}}=\int\nolimits _{-d/2}^{d/2}exp\left[-\theta {\left(t-\frac{{\beta}_{n}-{\delta}_{i}}{2\theta}\right)}^{2}\right]\left(-1\right)\left(-t+\frac{{\beta}_{n}-{\delta}_{i}}{2\theta}\right)dt$$

#### Second derivatives of the log-likelihood function

41 $$\begin{array}{lc}\frac{{\partial}^{2}ln{f}^{\ast}\left({\beta}_{n}|{\mathbf{X}}_{n}\right)}{\partial {\beta}_{n}^{2}}=\frac{{\partial}^{2}ln\left[P\left({\mathbf{X}}_{n}|{\beta}_{n}\right)\right]}{\partial {\beta}_{n}^{2}}+\frac{{\partial}^{2}ln\left[g\left(\beta \right)\right]}{\partial {\beta}_{n}^{2}}\\ \ \ \ \ \ \ \ \ \ \ \ \ \ \ \ =\sum\limits_{\mathrm{i}=1}^{\mathrm{I}}\frac{{\partial}^{2}}{\partial {\beta}_{n}^{2}}lnf\left({x}_{ni}\vert\Theta \right)-\frac{1}{{\sigma}^{2}}\end{array}$$

42 $$\begin{array}{lc}\frac{{\partial}^{2}ln{f}^{\ast}\left({\beta}_{n}|{\mathbf{X}}_{n}\right)}{\partial {\beta}_{n}\partial {\beta}_{m}}=\frac{{\partial}^{2}ln\left[P\left({\mathbf{X}}_{n}|{\beta}_{n}\right)\right]}{\partial {\beta}_{n}\partial {\beta}_{m}}+\frac{{\partial }^{2}ln\left[g\left({\beta}_{n}\right)\right]}{\partial {\beta}_{n}\partial {\beta}_{m}}\\ \ \ \ \ \ \ \ \ \ \ \ \ \ \ \ =\sum\limits_{\mathrm{i}=1}^{\mathrm{I}}\frac{{\partial}^{2}}{\partial {\beta }_{n}\partial {\beta }_{m}}lnf\left({x}_{ni}|\Theta \right)+\frac{\partial }{\partial {\beta}_{m}}\left(-\frac{{\beta}_{n}-\mu}{{\sigma}^{2}}\right)\\ \ \ \ \ \ \ \ \ \ \ \ \ \ \ \ =0\end{array}$$

where

43 $$\frac{{\partial}^{2}}{\partial {\beta}_{n}^{2}}lnf\left({x}_{ni}|\Theta \right)=\frac{\partial}{\partial {\beta}_{n}}\left({A}_{\beta}^{\prime}-\frac{{\gamma}_{\beta}^{\prime}}{\gamma}\right)={A}_{\beta}^{^{\prime\prime}}-\frac{{\gamma}_{\beta}^{^{\prime\prime}}\gamma-{{\gamma}^{\prime}}_{\beta}^{2}}{{\gamma}^{2}}$$

44 $${A}_{\beta}^{^{\prime\prime}}=\frac{\partial}{\partial {\beta}_{n}}\left[-\left(-{x}_{ni}+\frac{{\beta}_{n}-{\delta}_{i}}{2\theta}\right)\right]=-\frac{1}{2\theta}$$

45 $${\gamma}_{\beta}^{^{\prime\prime}}=\frac{\partial {\gamma}_{\beta}^{\prime}}{\partial {\beta}_{n}}=\int\nolimits_{-d/2}^{d/2}exp\left[-\theta {\left(t-\frac{{\beta }_{n}-{\delta }_{i}}{2\theta }\right)}^{2}\right]\left[{\left(-t+\frac{{\beta }_{n}-{\delta }_{i}}{2\theta }\right)}^{2}-\frac{1}{2\theta }\right]dt$$

### Derivatives of the log-likelihood function with respect to the dispersion parameter

#### First derivatives of the log-likelihood function

46 $$\begin{array}{lc}\frac{\partial lnP\left(\mathbf{X}\vert\Theta \right)}{\partial \theta}=\sum\limits_{n=1}^{N}\sum\limits_{i=1}^{I}\frac{\partial}{\partial \theta}lnf\left({x}_{ni}\vert\Theta \right)\\ \ \ \ \ \ \ \ \ \ \ \ \ =\sum\limits_{n=1}^{N}\sum\limits_{i=1}^{I}\frac{\partial}{\partial \theta}\left(ln\left({e}^{A}\right)-ln\gamma \right)\\ \ \ \ \ \ \ \ \ \ \ \ \ =\sum\limits_{n=1}^{N}\sum\limits_{i=1}^{I}{A}_{\theta}^{\prime}-\frac{{\gamma }_{\theta}^{\prime}}{\gamma}\end{array}$$

where

47 $$A=-\theta {\left({x}_{ni}-\frac{{\beta }_{n}-{\delta }_{i}}{2\theta }\right)}^{2}$$

48 $${A}_{\theta}^{\prime}=\frac{\partial }{\partial \theta}\left[-\theta {\left({x}_{ni}-\frac{{\beta}_{n}-{\delta}_{i}}{2\theta}\right)}^{2}\right]=-{x}_{ni}^{2}+{\left(\frac{{\beta}_{n}-{\delta}_{i}}{2\theta}\right)}^{2}$$

49 $${\gamma}_{\theta}^{\prime}=\frac{\partial \gamma }{\partial \theta}=\int\nolimits _{-d/2}^{d/2}exp\left[-\theta {\left(t-\frac{{\beta}_{n}-{\delta}_{i}}{2\theta}\right)}^{2}\right]\left(-{t}^{2}+{\left(\frac{{\beta}_{n}-{\delta}_{i}}{2\theta}\right)}^{2}\right)dt$$

#### Second derivatives of the log-likelihood function

50 $$\begin{array}{lc}\frac{{\partial}^{2}lnP\left(\mathbf{X}|\Theta \right)}{\partial {\theta}^{2}}=\sum\limits_{n=1}^{N}\sum\limits_{i=1}^{I}\frac{{\partial}^{2}}{\partial {\theta}^{2}}lnf\left({x}_{ni}|\Theta \right)\\ \ \ \ \ \ \ \ \ \ \ \ \ \ =\sum\limits_{n=1}^{N}\sum\limits_{i=1}^{I}\frac{\partial}{\partial \theta}\left({A}_{\theta}^{\prime}-\frac{{\gamma}_{\theta}^{\prime}}{\gamma}\right)\\ \ \ \ \ \ \ \ \ \ \ \ \ \ =\sum\limits_{n=1}^{N}\sum\limits_{i=1}^{I}{A}_{\theta}^{^{\prime\prime}}-\frac{{\gamma}_{\theta}^{^{\prime\prime}}\gamma -{{\gamma}^{\prime}}_{\theta}^{2}}{{\gamma}^{2}}\end{array}$$

where

51 $${A}_{\theta}^{^{\prime\prime}}=\frac{\partial}{\partial \theta}\left[-{x}_{ni}^{2}+{\left(\frac{{\beta}_{n}-{\delta}_{i}}{2\theta}\right)}^{2}\right]=-\frac{{\left({\beta}_{n}-{\delta}_{i}\right)}^{2}}{2{\theta}^{3}}$$

52 $${\gamma}_{\theta}^{^{\prime\prime} }=\frac{\partial {\gamma}_{\theta}^{\prime}}{\partial \theta}={\int}_{-d/2}^{d/2}exp\left[-\theta {\left(t-\frac{{\beta}_{n}-{\delta}_{i}}{2\theta}\right)}^{2}\right]\left[{\left(-{t}^{2}+{\left(\frac{{\beta}_{n}-{\delta}_{i}}{2\theta}\right)}^{2}\right)}^{2}-\frac{{\left({\beta}_{n}-{\delta}_{i}\right)}^{2}}{2{\theta}^{3}}\right]dt$$

## Appendix 3: Parameter recovery of the estimation algorithm for Verhelst's model

Appendix Table 10.

**Table 10:** Parameter recovery for person, item, and dispersion parameters

			Unit normal distribution with line length = 1		Unit normal distribution with line length = 5		Nonnormal distribution with line length = 5	
Parameter	Test length	Sample size	MAD	RMSE	MAD	RMSE	MAD	RMSE
Person ability	20	200	0.53	0.66	0.27	0.33	0.25	0.31
		500	0.49	0.61	0.25	0.31	0.28	0.36
		1000	0.48	0.60	0.25	0.31	0.27	0.34
	40	200	0.41	0.51	0.18	0.23	0.19	0.25
		500	0.42	0.52	0.18	0.22	0.18	0.23
		1000	0.41	0.51	0.18	0.23	0.19	0.25
	60	200	0.35	0.43	0.15	0.19	0.16	0.21
		500	0.35	0.43	0.15	0.19	0.16	0.20
		1000	0.34	0.42	0.15	0.19	0.15	0.20
Item difficulty	20	200	0.22	0.27	0.11	0.14	0.12	0.16
		500	0.14	0.17	0.06	0.08	0.10	0.13
		1000	0.10	0.13	0.05	0.06	0.08	0.10
	40	200	0.22	0.28	0.11	0.14	0.13	0.17
		500	0.15	0.18	0.07	0.08	0.08	0.10
		1000	0.10	0.12	0.05	0.06	0.06	0.08
	60	200	0.23	0.28	0.11	0.14	0.12	0.16
		500	0.14	0.18	0.07	0.09	0.08	0.10
		1000	0.10	0.13	0.05	0.06	0.06	0.08
Item-level dispersion	20	200	0.68	0.85	0.09	0.11	0.08	0.10
		500	0.45	0.56	0.06	0.07	0.08	0.09
		1000	0.34	0.43	0.05	0.06	0.06	0.08
	40	200	0.63	0.82	0.08	0.10	0.08	0.10
		500	0.47	0.58	0.05	0.06	0.05	0.06
		1000	0.34	0.42	0.04	0.05	0.05	0.06
	60	200	0.67	0.86	0.08	0.10	0.09	0.11
		500	0.47	0.59	0.05	0.06	0.06	0.07
		1000	0.34	0.42	0.04	0.05	0.04	0.05

## Code availability

The program code used in this research is available in the Open Science Framework repository at https://osf.io/c3jzy/?view_only=0314d5f0cbef4627812ac0bf5d54d271.

## References

[CR1] Adams, R. J., Wu, M. L., Cloney, D., Berezner, A., & Wilson, M. R. (2020). *ACER ConQuest: Generalised item response modelling software (Version 5.29) [Computer software]*. Australian Council for Educational Research. https://www.acer.org/au/conquest. Accessed 25 Feb 2021

[CR2] Andersen, E. B. (1970). Asymptotic properties of conditional maximum likelihood estimators. *Journal of the Royal Statistical Society. Series B, Statistical Methodology,**32*(2), 283–301.

[CR3] Andrich, D. (1978). A rating formulation for ordered response categories. *Psychometrika,**43*(4), 561–573. 10.1007/BF02293814

[CR4] Andrich, D. (1982). An extension of the Rasch model for ratings providing both location and dispersion parameters. *Psychometrika,**47*(1), 105–113. 10.1007/bf02293856

[CR5] Baker, F. B., & Kim, S.-H. (2004).* Item response theory: Parameter estimation techniques* (2nd ed.). Marcel Dekker.

[CR6] Barrows, P. D., & Thomas, S. A. (2018). Assessment of mood in aphasia following stroke: Validation of the dynamic visual analogue mood scales (D-VAMS). *Clinical Rehabilitation,**32*(1), 94–102. 10.1177/0269215517714590 28653547 10.1177/0269215517714590

[CR7] Bock, R. D., & Aitkin, M. (1981). Marginal maximum likelihood estimation of item parameters: Application of an EM algorithm. *Psychometrika,**46*(4), 443–459.

[CR8] Bond, T. G., & Fox, C. M. (2015). *Applying the**Rasch**model: Fundamental measurement in the human sciences* (3rd ed.). Routledge. 10.4324/9781315814698

[CR9] Brown, A., & Maydeu-Olivares, A. (2011). Item response modeling of forced-choice questionnaires. *Educational and Psychological Measurement,**71*(3), 460–502. 10.1177/0013164410375112

[CR10] Cai, L., Thissen, D., & du Toit, S. H. C. (2011). *IRTPRO: Flexible, multidimensional, multiple categorical IRT modeling* [Computer software]. Scientific Software International.

[CR11] Chimi, C. J., & Russell, D. L. (2009). *The Likert scale: A proposal for improvement using quasi-continuous variables [Paper presentation]*. ISECON.

[CR12] Chiu, C. K., & Alliger, G. M. (1990). A proposed method to combine ranking and graphic rating in performance appraisal: The quantitative ranking scale. *Educational and Psychological Measurement,**50*(3), 493–503.

[CR13] Choi, Y. J., & Asilkalkan, A. (2019). R packages for item response theory analysis: Descriptions and features. *Measurement: Interdisciplinary Research And Perspectives,**17*(3), 168–175. 10.1080/15366367.2019.1586404

[CR14] Choppin, B. (1985). A fully conditional estimation procedure for Rasch model parameters. *Evaluation in Education,**9*(1), 29–42.

[CR15] Cook, C., Heath, F., Thompson, R. L., & Thompson, B. (2001). Score reliability in web or internet-based surveys: Unnumbered graphic rating scales versus Likert-type scales. *Educational and Psychological Measurement,**61*(4), 697–706. 10.1177/00131640121971356

[CR16] Couper, M. P., Tourangeau, R., Conrad, F. G., & Singer, E. (2006). Evaluating the effectiveness of visual analog scales: A web experiment. *Social Science Computer Review,**24*(2), 227–245. 10.1177/0894439305281503

[CR17] Cox, E. P., III. (1980). The optimal number of response alternatives for a scale: A review. *Journal of Marketing Research,**17*(4), 407–422.

[CR18] Cui, C., Wang, C., & Xu, G. (2024). Variational estimation for multidimensional generalized partial credit model. *Psychometrika,**89*(3), 929–957. 10.1007/s11336-024-09955-8 38429494 10.1007/s11336-024-09955-8

[CR19] de Ayala, R. J. (2009). *The theory and practice of item response theory*. Guilford Press.

[CR20] de Leeuw, J., & Mair, P. (2007). An introduction to the special volume on “Psychometrics in R.” *Journal of Statistical Software,**20*(1), 1–5. 10.18637/jss.v020.i01

[CR21] Dumenci, L., & Achenbach, T. M. (2008). Effects of estimation methods on making trait-level inferences from ordered categorical items for assessing psychopathology. *Psychological Assessment,**20*(1), 55–62. 10.1037/1040-3590.20.1.55 18315399 10.1037/1040-3590.20.1.55

[CR22] Embretson, S. E. (1996). Item response theory models and spurious interaction effects in factorial ANOVA designs. *Applied Psychological Measurement,**20*(3), 201–212. 10.1177/014662169602000302

[CR23] Embretson, S. E., & Reise, S. P. (2000). *Item response theory for psychologists*. Erlbaum.

[CR24] Feng, Y., Parkin, D., & Devlin, N. J. (2014). Assessing the performance of the EQ-VAS in the NHS PROMs programme. *Quality Of Life Research,**23*(3), 977–989. 10.1007/s11136-013-0537-z 24081873 10.1007/s11136-013-0537-z PMC4287662

[CR25] Ferrando, P. J. (2001). A nonlinear congeneric model for continuous item responses. *British Journal of Mathematical and Statistical Psychology,**54*(2), 293–313. 10.1348/000711001159573 11817095 10.1348/000711001159573

[CR26] Fleishman, A. I. (1978). A method for simulating non-normal distributions. *Psychometrika,**43*(4), 521–532. 10.1007/BF02293811

[CR27] Flynn, D., Van Schaik, P., & Van Wersch, A. (2004). A comparison of multi-item Likert and visual analogue scales for the assessment of transactionally defined coping function. *European Journal of Psychological Assessment,**20*(1), 49–58. 10.1027/1015-5759.20.1.49

[CR28] García-Pérez, M. A. (2024). Are the steps on Likert scales equidistant? Responses on visual analog scales allow estimating their distances. *Educational and Psychological Measurement,**84*(1), 91–122. 10.1177/00131644231164316 38250504 10.1177/00131644231164316 PMC10795572

[CR29] Garner, M., & Engelhard, G. (2002). An eigenvector method for estimating item parameters of the dichotomous and polytomous Rasch models. *Journal of Applied Measurement,**3*(2), 107–128. 12011497

[CR30] Harwell, M. R., & Gatti, G. G. (2001). Rescaling ordinal data to interval data in educational research. *Review of Educational Research,**71*(1), 105–131. 10.3102/00346543071001105

[CR31] Hauser, K., & Walsh, D. (2008). Visual analogue scales and assessment of quality of life in cancer. *The Journal of Supportive Oncology,**6*(6), 277–282. 18724538

[CR32] Hayes, M. H., & Patterson, D. G. (1921). Experimental development of the graphic rating method. *Psychological Bulletin,**18*, 98–99.

[CR33] Hofmans, J., & Theuns, P. (2008). On the linearity of predefined and self-anchoring visual analogue scales. *The British Journal of Mathematical and Statistical Psychology,**61*(2), 401–413. 10.1348/000711007X206817 17535485 10.1348/000711007X206817

[CR34] Hohensinn, C. (2018). pcIRT: An R package for polytomous and continuous Rasch models. *Journal of Statistical Software,**84*(2), 1–14. 10.18637/jss.v084.c0230450020

[CR35] Hohensinn, C. (2019). pcIRT: IRT models for polytomous and continuous item responses. (R package Version 0.2.4). Retrieved from https://cran.r-project.org/web/packages/pcIRT/index.html. Accessed 17 Feb 2021

[CR36] Holland, J. L. (1997). *Making vocational choices: A theory of vocational personalities and work environments* (3rd ed.). Psychological Assessment Resources.

[CR37] Humphry, S. M. (2008). Understanding the unit in the Rasch model. *Journal of Applied Measurement,**9*(3), 249–264. 18753694

[CR38] Iramaneerat, C., Smith, E., Jr., & Smith, R. M. (2008). An introduction to Rasch measurement. In J. Osborne (Ed.), *Best practices in Quantitative methods* (pp. 50–70). SAGE Publications. 10.4135/9781412995627

[CR39] Johnson, M. S. (2017). Bayesian inference using Gibbs sampling (BUGS) for IRT models. In W. J. van der Linden (Ed.), *Handbook of item response theory* (vol. 3, pp. 421–434). Chapman and Hall/CRC.

[CR40] Kersten, P., White, P. J., & Tennant, A. (2014). Is the pain visual analogue scale linear and responsive to change? An exploration using Rasch analysis. *PLoS One,**9*(6), Article e99485. 10.1371/journal.pone.0099485 24921952 10.1371/journal.pone.0099485 PMC4055724

[CR41] Krieg, E. F. (1999). Biases induced by coarse measurement scales. *Educational and Psychological Measurement,**59*(5), 749–766. 10.1177/00131649921970125

[CR42] Kuhlmann, T., Dantlgraber, M., & Reips, U. D. (2017). Investigating measurement equivalence of visual analogue scales and Likert-type scales in internet-based personality questionnaires. *Behavior Research Methods,**49*(6), 2173–2181. 10.3758/s13428-016-0850-x 28130728 10.3758/s13428-016-0850-x

[CR43] Li, C. H. (2016). Confirmatory factor analysis with ordinal data: Comparing robust maximum likelihood and diagonally weighted least squares. *Behavior Research Methods,**48*(3), 936–949. 10.3758/s13428-015-0619-7 26174714 10.3758/s13428-015-0619-7

[CR44] Linacre, J. M. (2023). *Winsteps**Rasch measurement computer program* (Version 5.6.0) [Computer software]. Winsteps.com. https://www.winsteps.com/

[CR45] May, T., & Pridmore, S. (2020). A visual analogue scale companion for the six-item Hamilton Depression Rating Scale. *Australian Psychologist,**55*(1), 3–9. 10.1111/ap.12427

[CR46] Mellenbergh, G. J. (1994). A unidimensional latent trait model for continuous item responses. *Multivariate Behavioral Research,**29*(3), 223–236. 10.1207/s15327906mbr2903_2 26765136 10.1207/s15327906mbr2903_2

[CR47] Molenaar, D., Cúri, M., & Bazán, J. L. (2022). Zero and one inflated item response theory models for bounded continuous data. *Journal of Educational and Behavioral Statistics,**47*(6), 693–735. 10.3102/10769986221108455

[CR48] Müller, H. (1987). A rasch model for continuous ratings. *Psychometrika,**52*(2), 165–181. 10.1007/BF02294232

[CR49] Müller, H. (1999). *Probabilistische Testmodelle fuer diskrete und kontinuierliche Ratingskalen [Probabilistic models for discrete and continuous rating scales]*. Huber.

[CR50] Noel, Y., & Dauvier, B. (2007). A beta item response model for continuous bounded responses. *Applied Psychological Measurement,**31*(1), 47–73. 10.1177/0146621605287691

[CR51] Pfennings, L., Cohen, L., & van der Ploeg, H. (1995). Preconditions for sensitivity in measuring change: Visual analogue scales compared to rating scales in a Likert format. *Psychological Reports,**77*(2), 475–480. 10.2466/pr0.1995.77.2.475 8559872 10.2466/pr0.1995.77.2.475

[CR52] Price, D. D., McGrath, P. A., Rafii, A., & Buckingham, B. (1983). The validation of visual analogue scales as ratio scale measures for chronic and experimental pain. *Pain,**17*(1), 45–56. 10.1016/0304-3959(83)90126-4 6226917 10.1016/0304-3959(83)90126-4

[CR53] Reips, U.-D., & Funke, F. (2008). Interval-level measurement with visual analogue scales in internet-based research: VAS generator. *Behavior Research Methods,**40*(3), 699–704. 10.3758/BRM.40.3.699 18697664 10.3758/brm.40.3.699

[CR54] Rijmen, F. (2009). Efficient full information maximum likelihood estimation for multidimensional IRT models. *ETS Research Report Series,**2009*(1), i–31. 10.1002/j.2333-8504.2009.tb02160.x

[CR55] Roberts, J. S., Donoghue, J. R., & Laughlin, J. E. (2000). A general item response theory model for unfolding unidimensional polytomous responses. *Applied Psychological Measurement,**24*(1), 3–32. 10.1177/01466216000241001

[CR56] Robitzsch, A. (2024). sirt: Supplementary item response theory models (R package Version 4.1-15). Retrieved from https://cran.r-project.org/web/packages/sirt/index.html

[CR57] Russell, C. J., & Bobko, P. (1992). Moderated regression analysis and Likert scales: Too coarse for comfort. *Journal of Applied Psychology,**77*(3), 336–342. 10.1037/0021-9010.77.3.336 1601825 10.1037/0021-9010.77.3.336

[CR58] Samejima, F. (1973). Homogeneous case of the continuous response model. *Psychometrika,**38*(2), 203–219. 10.1007/bf02291114

[CR59] Simms, L. J., Zelazny, K., Williams, T. F., & Bernstein, L. (2019). Does the number of response options matter? Psychometric perspectives using personality questionnaire data. *Psychological Assessment,**31*(4), 557–556. 10.1037/pas0000648 30869956 10.1037/pas0000648

[CR60] Smith, R. M. (2000). Fit analysis in latent trait measurement models. *Journal of Applied Measurement,**1*(2), 199–218. 12029178

[CR61] Spiegelhalter, D. J., Thomas, A., & Best, N. (2003). *WinBUGS (Version 1.4) [Computer program]*. MRC Biostatistics Unit.

[CR62] Sung, Y.-T., & Wu, J.-S. (2018). The visual analogue scale for rating, ranking and paired-comparison (VAS-RRP): A new technique for psychological measurement. *Behavior Research Methods,**50*(4), 1694–1715. 10.3758/s13428-018-1041-8 29667082 10.3758/s13428-018-1041-8 PMC6096654

[CR63] Sung, Y.-T., Cheng, Y.-W., & Wu, J.-S. (2016). Constructing a situation-based career interest assessment for junior high school students and examining their interest structure. *Journal of Career Assessment,**24*(2), 347–365. 10.1177/1069072715580419

[CR64] Sung, Y.-T., Cheng, Y.-W., & Hsueh, J.-H. (2017). Identifying the career-interest profiles of junior-high-school students through latent profile analysis. *The Journal of Psychology,**151*(3), 229–246. 10.1080/00223980.2016.1261076 27982740 10.1080/00223980.2016.1261076

[CR65] Sung, Y.-T., Chang, Y.-T.Y., Cheng, T.-Y., & Tien, H.-L.S. (2019). Development and validation of a work values scale for assessing high school students: A mixed methods approach. *European Journal of Psychological Assessment,**35*(4), 526–543.

[CR66] Tourangeau, R., Rips, L. J., & Rasinski, K. (2000). *The Psychology of Survey Response*. Cambridge University Press.

[CR67] Verhelst, N. D. (2019). Exponential family models for continuous responses. In B. P. Veldkamp & C. Sluijter (Eds.), *Theoretical and Practical Advances in Computer-based Educational Measurement* (pp. 135–160). Springer. 10.1007/978-3-030-18480-3_7

[CR68] Wang, T., & Zeng, L. (1998). Item parameter estimation for a continuous response model using an EM algorithm. *Applied Psychological Measurement,**22*(4), 333–344. 10.1177/014662169802200402

[CR69] Wright, B. D., & Linacre, J. M. (1994). Reasonable mean-square fit values. *Rasch Measurement Transactions,**8*(3), 370–371.

[CR70] Wright, B., & Masters, G. (1982). *Rating scale analysis*. MESA Press.

[CR71] Wright, B. D., & Mok, M. M. C. (2004). An overview of the family of Rasch measurement models. In E. V. Smith & R. M. Smith (Eds.), *Introduction to Rasch measurement: Theory, models and applications* (pp. 1–24). JAM Press.

[CR72] Zopluoglu, C. (2012). EstCRM: An R package for Samejima’s continuous IRT model. *Applied Psychological Measurement,**36*(2), 149–150.

