# Supplementary material for: Continuous Rating Scale Analytics (CoRSA): A tool for analyzing continuous and discrete data with item response theory
Source: Behav Res Methods. 2025 Nov 4;57(12):333. doi: 10.3758/s13428-025-02848-3 (PMC12586417; doi:10.3758/s13428-025-02848-3)
Supplement: Supplementary file 1 — Supplementary file1 (ZIP 1608 KB) [file 13428_2025_2848_MOESM1_ESM.zip › CoRSATutorialSupplmentData_V3/An worked example of conducting CoRSM analysis using CoRSA on the VSA-RRP 2.0 platform.pdf]

Conducting CoRSM Analysis Using CoRSA on the VAS-RRP 2.0 Platform

This example demonstrates how to perform a continuous rating scale model (CoRSM; Müller, 1987) analysis using CoRSA via the VAS-RRP 2.0 platform.

Step 1: Set Up Variables for CoRSM Analysis Using the CoRSA Interface

1. Visit http://vasrrp.net/vasrrp2.

   Users can learn more about "**What is CoRSA**" by clicking the Continuous Rating Scale Analytics (CoRSA) icon or by scrolling to the bottom of the homepage.

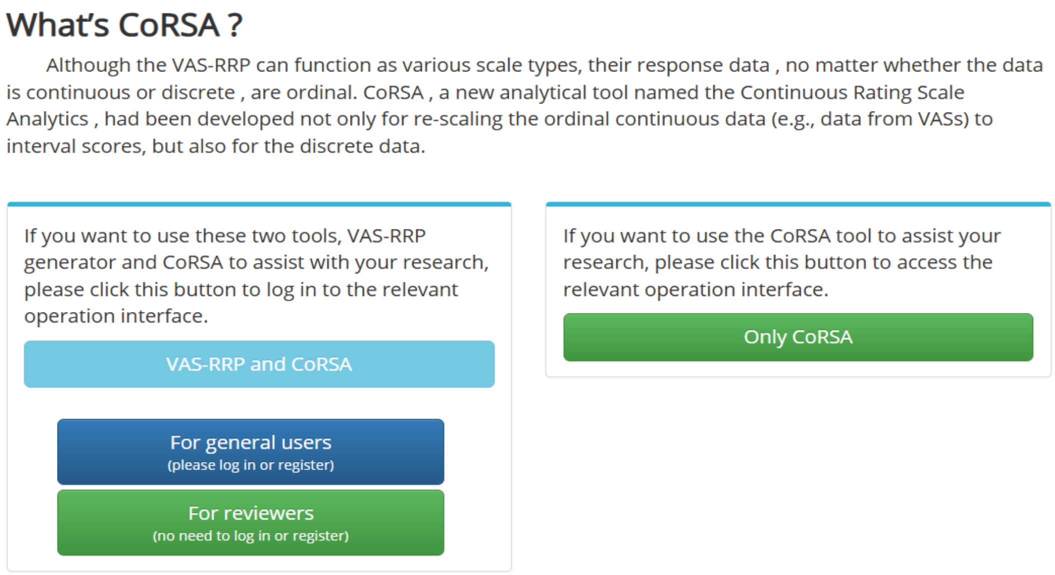

2. Select "**For general users**", then either register for an account or log in your existing account. After logging in, the main interface will appear as shown below:

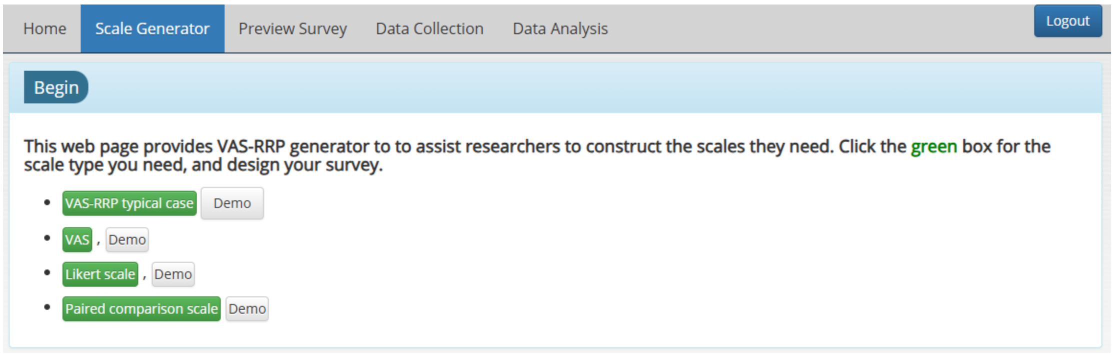

3. Select "**Data Analysis**" and follow these steps:

(1) Choose your response scale type:

Select either "**Discrete scale**" or "**Continuous scale**" depending on the type of response scale in your dataset. For example, if your data were collected from a visual analogue scale (VAS), choose "**Continuous scale**". Once selected, the interface will appear as shown below:

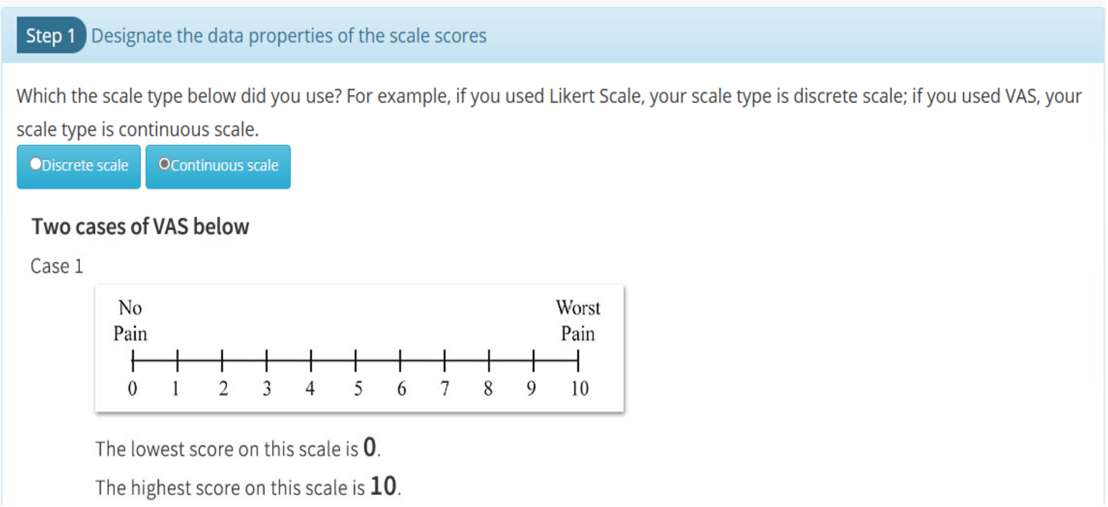

(2) Specify score range:

Enter the **lowest** and **highest** possible values of your response scale. These values are required for CoRSA to conduct the subsequent analysis. For example, the example dataset (downloaded in Step 2) uses a score range from -3.00 to 3.00. Users should enter -3.00 and 3.00 respectively in the input fields.

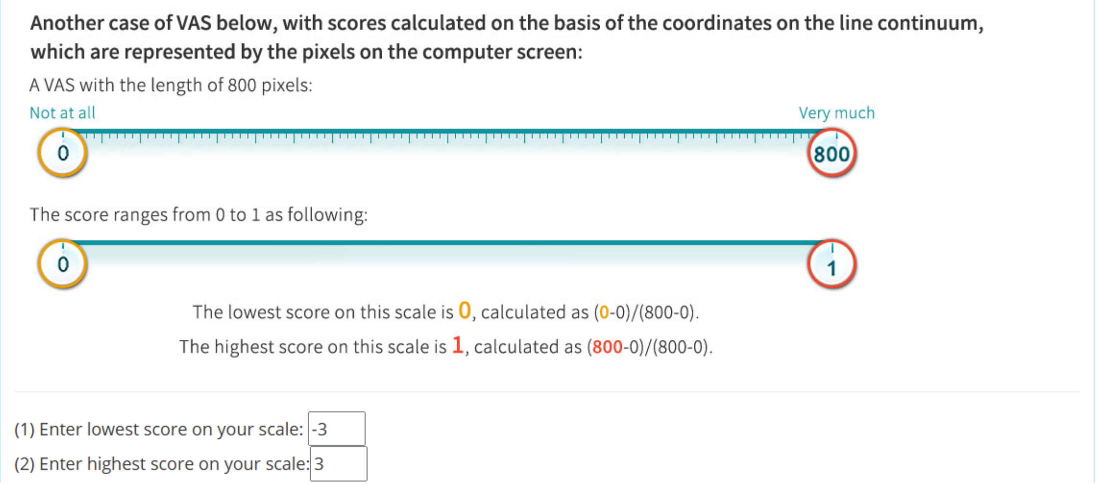

(3) Specify the analytical model:

Select either the one-parameter or two-parameter model for analyzing your dataset. The continuous rating scale model proposed by Müller (1987) is referred to as the **one-parameter model** here, as it includes one difficulty parameter per item. Verhelst (2019) extended Müller’s model by incorporating both item difficulty and item-level dispersion for each item, which is referred to as the **two-parameter model** in this context.

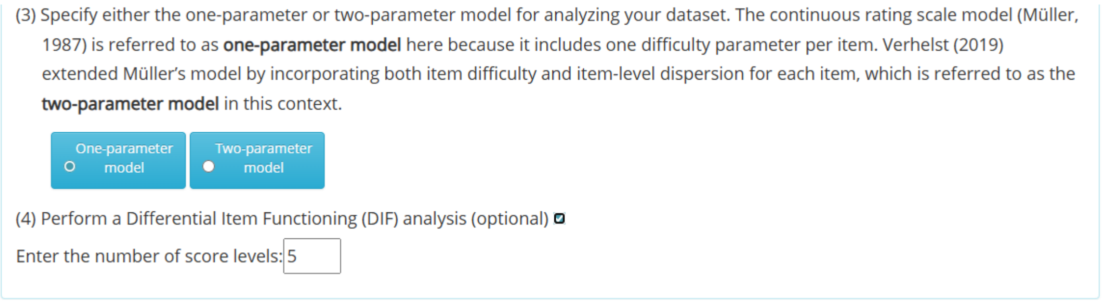

(4) (Optional) Enable DIF analysis:

If you wish to conduct a differential item functioning (DIF) analysis, check the "**Perform a DIF analysis**" option and specify the **number of score levels** to divide the sample (the default is **5**). This step is optional—users can still perform CoRSM estimation even if " **Perform a DIF analysis** " option is not checked.

Step 2: Download the Template to Prepare Your Data

1. Click the button "**Download the template of the data file with DIF**". A CSV file will be downloaded to your device.

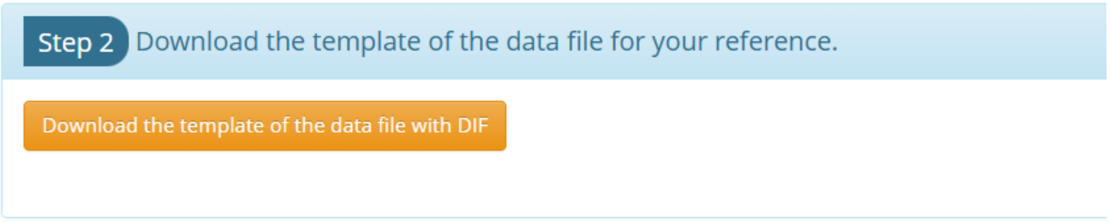

2. Open the downloaded CSV file. The file provides an example dataset consisting of 101 rows and 12 columns:

- The first row contains the variable names.

- The first column contains respondent IDs.
- The second column contains the grouping variable (e.g., 0 for female, 1 for male).
- The third to twelfth columns represent responses to Item 1 to Item 10, respectively.
- The second to 101st rows correspond to responses from individual respondents.

Example:

Person ID	Group	Item1	Item2	…	Item10
id1	1	-1.009	-1.991	…	-0.007
id2	1	0.604	-0.484	…	1.436
…	…	…	…	…	…
id 51	2	-3.000	-3.000	…	0.931
id 52	2	-0.824	0.092	…	0.206
…	…	…	…	…	…

3. The response values can be:

- Continuous scores ranging from 0 to 10, such as data collected via visual analogue scales (VAS);
- Continuous scores ranging from 0 to 800, such as those from VAS-RRP (Sung & Wu, 2018); or
- Transformed scores derived from these continuous response scales.

Step 3: Upload Your CSV File and Download the Output

1. Click the "**Choose File**" button and select your prepared CSV data file from your device. Once the file is selected, press the “OK” button to confirm.

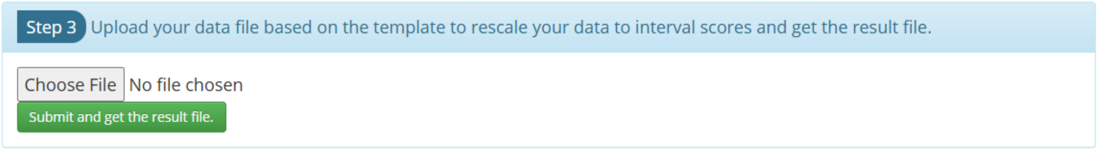

2. Click the "**Submit and get the result file**" button. The CoRSA system will immediately begin the parameter estimation process.
3. Once the estimation is complete, an output file containing the analysis results will be automatically downloaded to your device.

Step 4: Review the Output

1. Example 1—The data were analyzed using the one-parameter model (Müller’s continuous rating scale model), and the "**Perform a DIF analysis**" option was not selected.

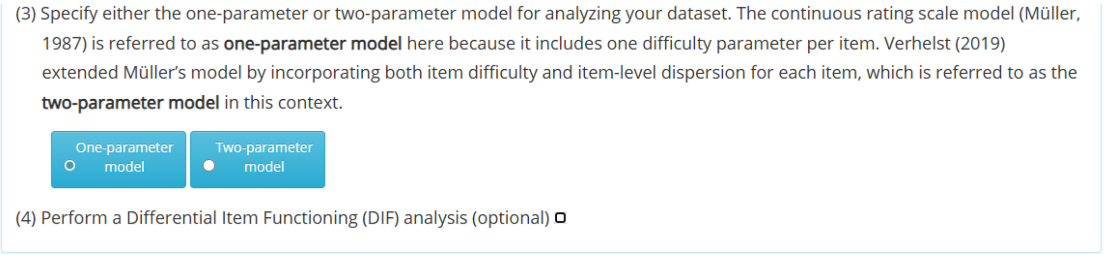

The output file will include the following results:

- **Person Parameter Estimates**
  A list of ability estimates for each respondent.
- **Item Parameter Estimates**
  A list of difficulty estimates and corresponding standard errors for each item.
- **Dispersion Estimate**
  The dispersion estimate for the overall test (or scale).

The figure below presented a sample output based on a dataset of 100 respondents and 10 items:

- The **upper section** of the output displayed descriptive statistics for the ability and difficulty estimates. For example, the mean, standard deviation, maximum, and minimum of the ability estimates were -0.015, 0.755, 2.569, and -2.069, respectively.
- The **lower section** of the output presented detailed information for each respondent and item. For example, the ability estimates for the first and second respondents was -0.537 and 0.555, respectively. The difficulty estimate and standard error for the first item were 0.237 and 0.094, while for the second item, they were 0.491 and 0.095. The test-level dispersion estimate was 0.358.

Descriptive statistics	person abilities		item difficulty		
mean	-0.015		0	0.094	
std	0.755		0.249	0.001	
max	2.569		0.491	0.095	
min	-2.069		-0.383	0.093	
The estimates of	person's abilities		item's difficulty		dispersion
person ID	estimates	Item ID	estimates	measurement errors	0.358
id 1	-0.537	item1	0.237	0.094	
id 2	0.555	item2	0.491	0.095	
id 3	-0.253	item3	-0.029	0.093	
id 4	0.982	item4	-0.037	0.093	
id 5	-0.048	item5	0.039	0.093	
id 6	0.018	item6	-0.13	0.093	
id 7	-0.022	item7	0.166	0.093	
id 8	-0.001	item8	-0.222	0.093	
id 9	0.734	item9	-0.383	0.094	
id 10	0.119	item10	-0.132	0.093	

2. Example 2—The data were analyzed using the two-parameter model (continuous rating scale model with item-level dispersion per item), and the "**Perform a DIF analysis**" option was selected.

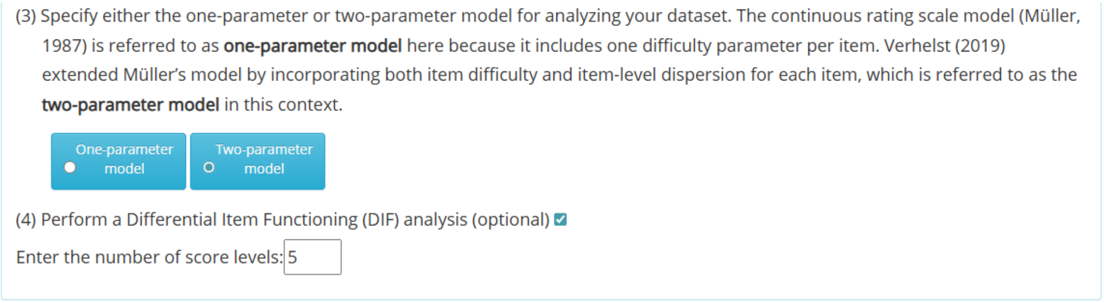

The output file will include the following results:

- **Person Parameter Estimates**
  A list of ability estimates for each respondent.
- **Item Parameter Estimates**
  A list of difficulty estimates and corresponding standard errors for each item.
- **Dispersion Estimate**
  A list of dispersion estimates for each item.

- **DIF Analysis**
  A list displaying the Mantel–Haenszel (MH) statistic, p-value, and effect size for each item.

The figure below presented a sample output based on a dataset of 100 respondents and 10 items:

- The **upper section** of the output displayed descriptive statistics for the ability and difficulty estimates. For example, the mean, standard deviation, maximum, and minimum of the ability estimates were -0.005, 0.774, 2.650, and -2.149, respectively.
- The **lower section** of the output presented detailed information for each respondent and item. For example, the ability estimates for the first and second respondents was -0.528 and 0.570, respectively. The difficulty estimate and standard error for the first item were 0.240 and 0.092, while for the second item, they were 0.445 and 0.089. The dispersion estimates for the first and second items was 0.333 and 0.276, respectively.
- In the **DIF result table**, the MH statistic, p-value, and effect size for the first item were 2.842, 0.092, and 0.107, respectively.

Descriptive statistics	person abilities		item difficulty					
mean	-0.005		0	0.096				
std	0.774		0.252	0.009				
max	2.65		0.445	0.106				
min	-2.149		-0.388	0.08				
The estimates of	person's abilities		item's difficulty		DIF			dispersion
person ID	estimates	Item ID	estimates	measurement errors	MH statistic	p-value	effect size	estimates
id 1	-0.528	item1	0.24	0.092	2.842	0.092	0.107	0.333
id 2	0.57	item2	0.445	0.089	1.885	0.17	0.017	0.276
id 3	-0.239	item3	-0.009	0.08	0.747	0.388	0.011	0.169
id 4	1.006	item4	-0.019	0.084	2.193	0.139	0.086	0.234
id 5	-0.034	item5	0.057	0.102	0.101	0.75	0.004	0.475
id 6	0.032	item6	-0.142	0.101	0.987	0.32	0.052	0.463
id 7	-0.008	item7	0.224	0.106	0.263	0.608	0.012	0.524
id 8	0.014	item8	-0.272	0.106	1.526	0.217	0.092	0.519
id 9	0.752	item9	-0.388	0.096	1.56	0.212	0.191	0.383
id 10	0.133	item10	-0.135	0.098	1.016	0.313	0.019	0.421
